# Supplementary figures and images for: Both pathogen and host dynamically adapt pH responses along the intestinal tract during enteric bacterial infection
Source: PLoS Biol. 2024 Aug 15;22(8):e3002761. doi: 10.1371/journal.pbio.3002761 (PMC11349234; doi:10.1371/journal.pbio.3002761)

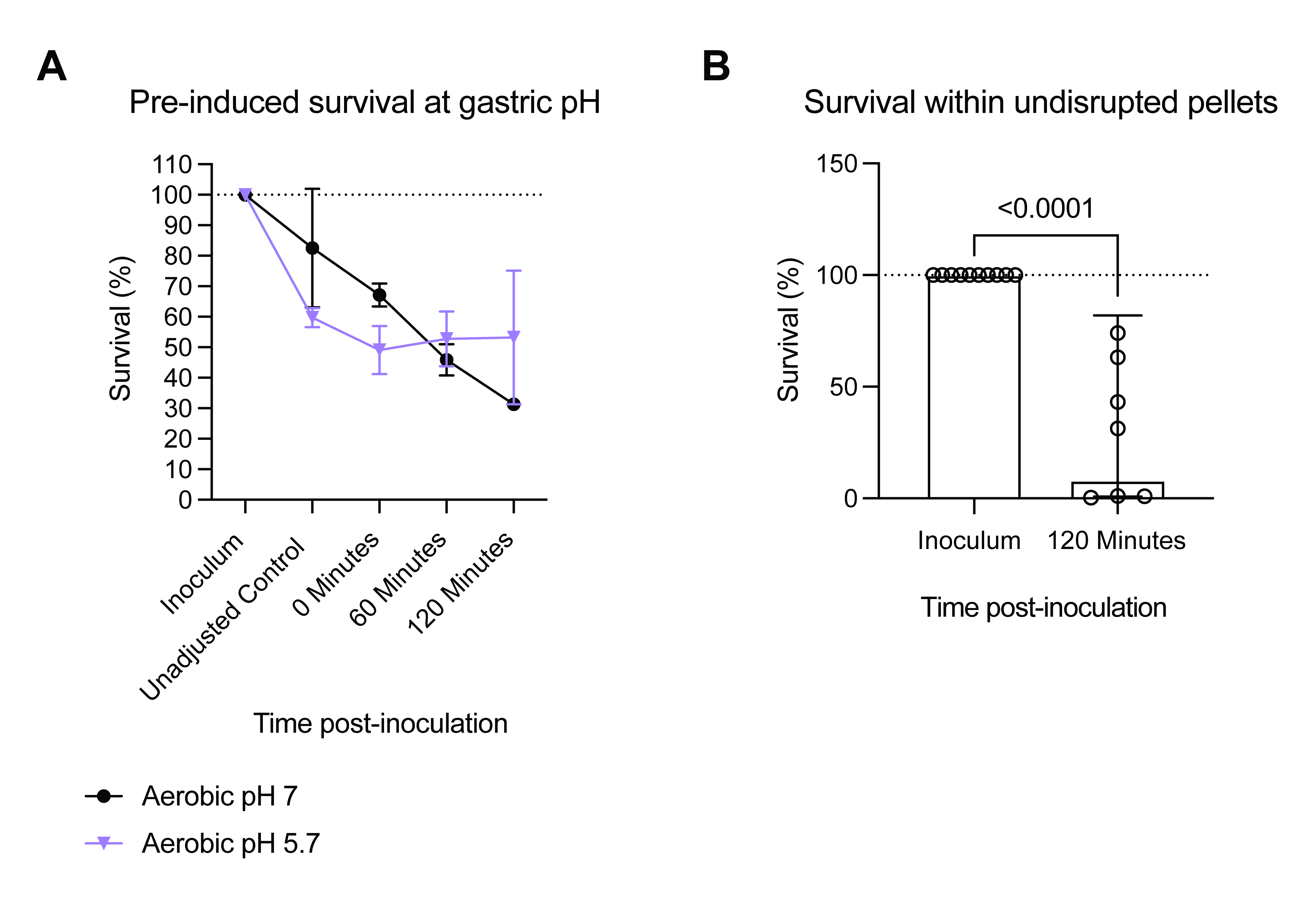

Supplement: S4 Fig — (A) Survival of C. rodentium pre-induced aerobically at either neutral pH 7 or colonic pH 5.7 for 3.5 h (to mid-log phase) before exposure to gastric pH 3.5. Each point represents the average of 3 biological replicates (3 technical replicates per biological replicate). Statistics represent a mixed-effects model with Geisser–Greenhouse correction and Šidák’s multiple comparisons test. Error represents mean +/− SD. (B) Survival of C. rodentium shed in the feces on day 2 postinfection (pi) at gastric pH 3.5. Fecal pellets were not disrupted before submersion in LB pH 3.5. Statistical analysis represents a Mann–Whitney test (N = 6–7). Summary data displayed in S4 Fig can be found in S1 Data. (TIF) [file pbio.3002761.s004.tif]

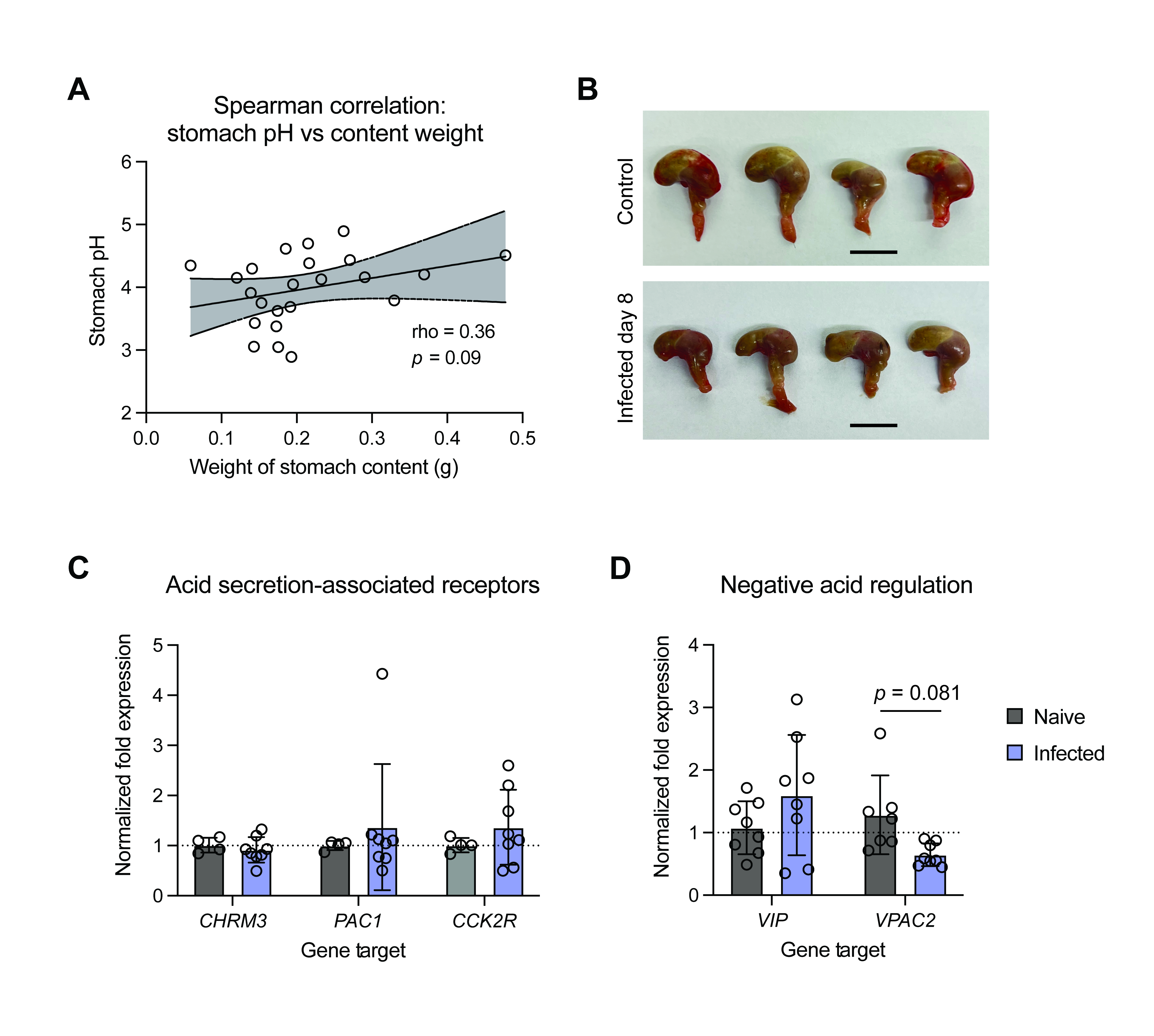

Supplement: S5 Fig — (A) Spearman correlation of stomach pH and weight of stomach contents (N = 23). Line represents linear regression +/− 95% confidence interval. p- and rho-values represent Spearman correlation values. (B) Representative stomach images from fasted control and day 8-infected mice. Scale bar represents 1 cm. (C) Normalized fold expression of genes associated with host regulation of acid secretion within stomach tissue from naïve and infected mice (N = 4–8). Statistics represent a two-way ANOVA with Šidák’s multiple comparisons test. (D) Normalized fold expression of genes associated with host negative regulation of acid secretion within stomach tissue from naïve and infected mice (N = 7–8). Statistics represent a two-way ANOVA with Šidák’s multiple comparisons test. Summary data displayed in S5 Fig can be found in S1 Data. (TIF) [file pbio.3002761.s005.tif]
