# Supplementary material for: Both pathogen and host dynamically adapt pH responses along the intestinal tract during enteric bacterial infection
Source: PLoS Biol. 2024 Aug 15;22(8):e3002761. doi: 10.1371/journal.pbio.3002761 (PMC11349234; doi:10.1371/journal.pbio.3002761)
Supplement: S1 Uncropped Images — (PDF) [file pbio.3002761.s008.pdf]

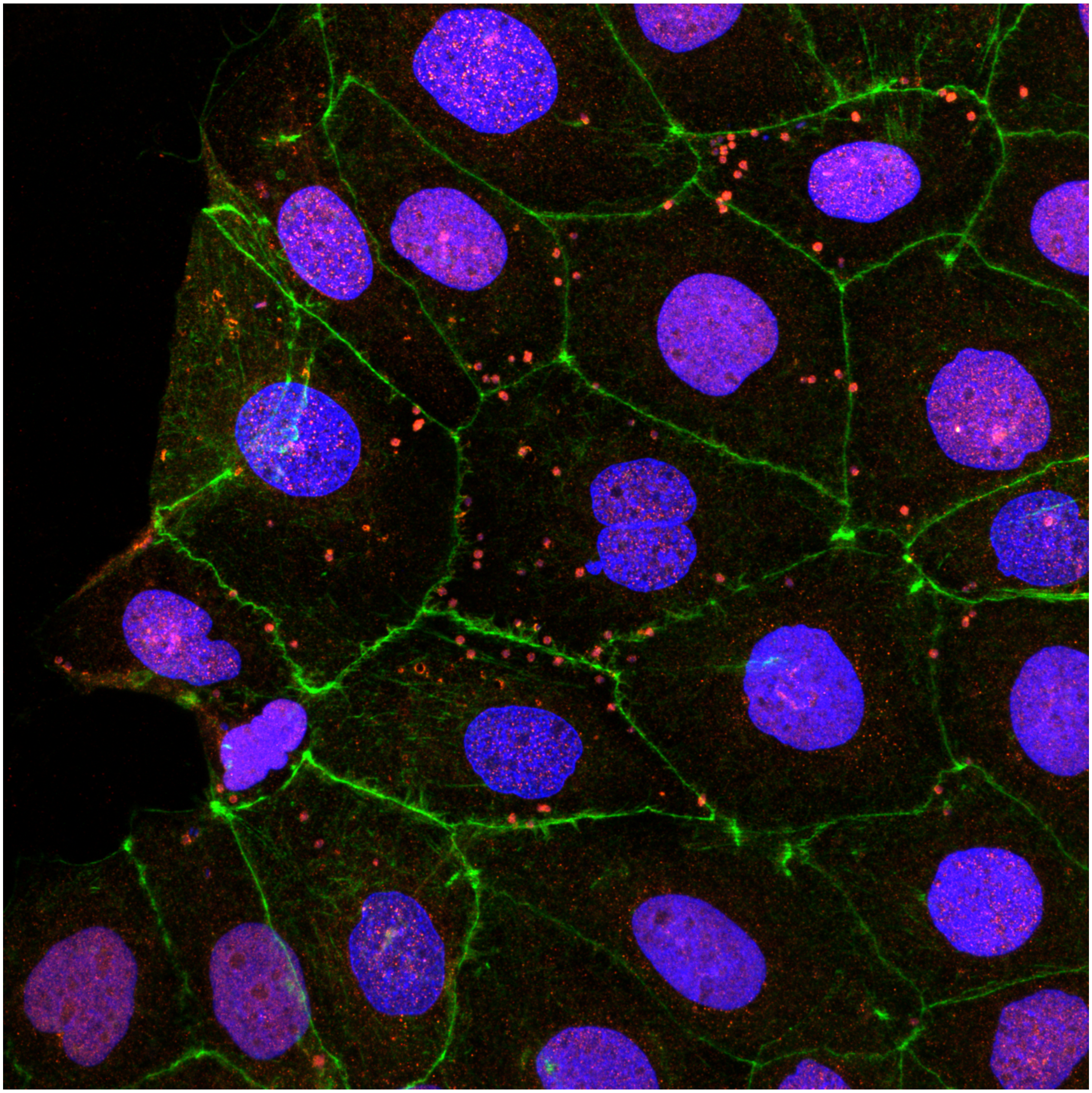

Caco-2/TC7 cells with pH 4.8 pre-induction Figure 2A - Replicate 1

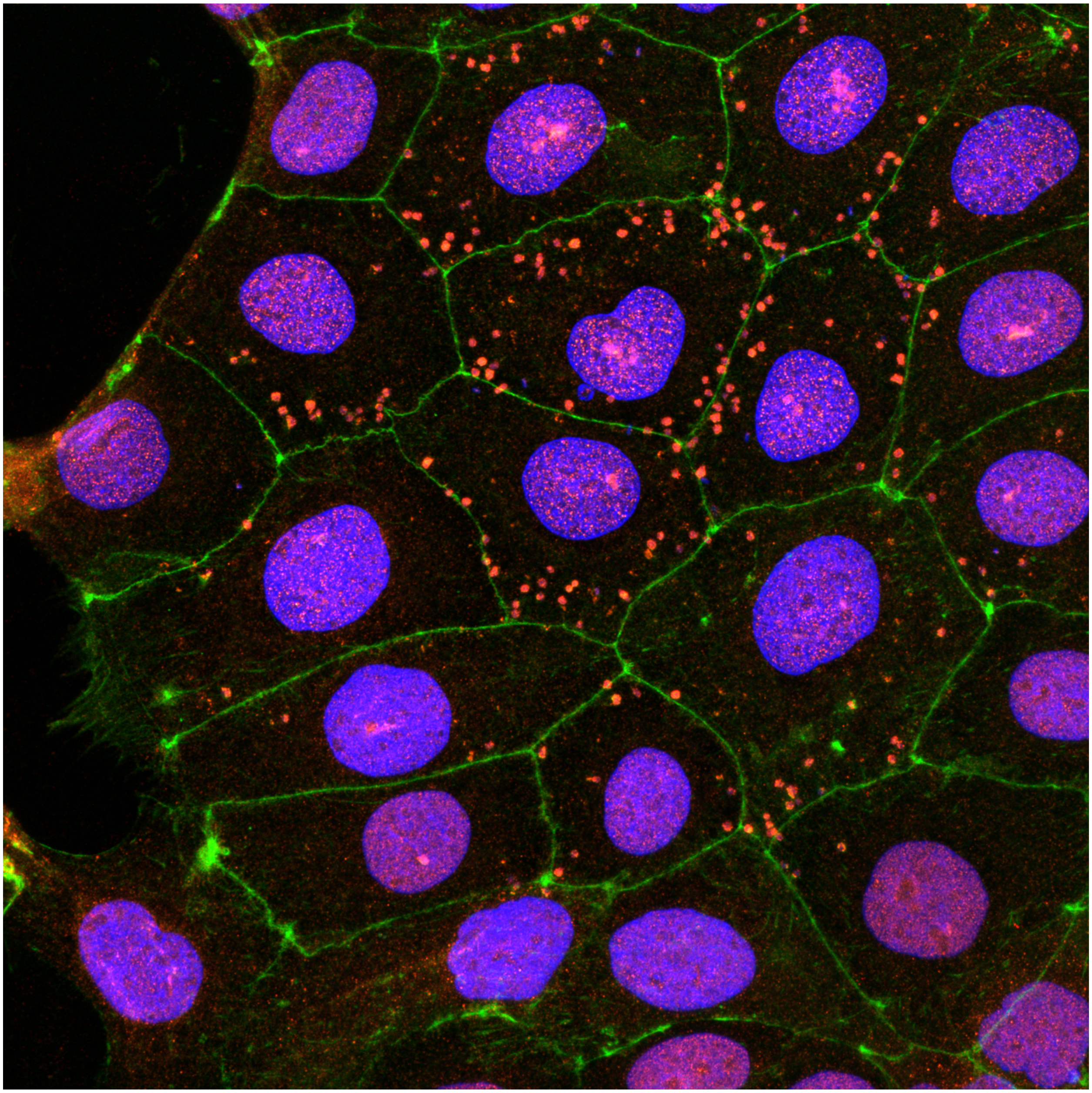

Caco-2/TC7 cells with pH 4.8 pre-induction Figure 2A - Replicate 2

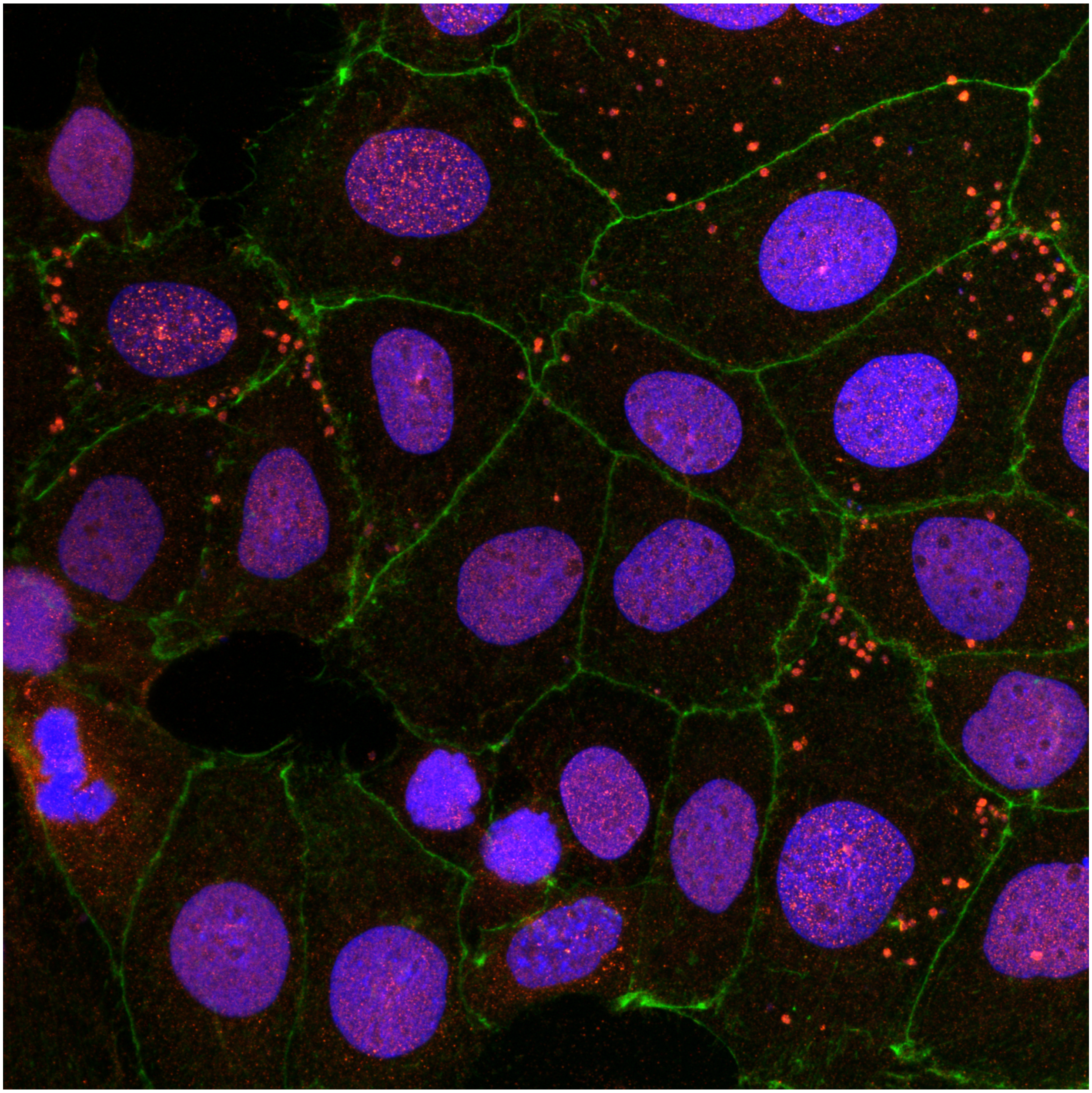

Caco-2/TC7 cells with pH 4.8 pre-induction Figure 2A - Replicate 3

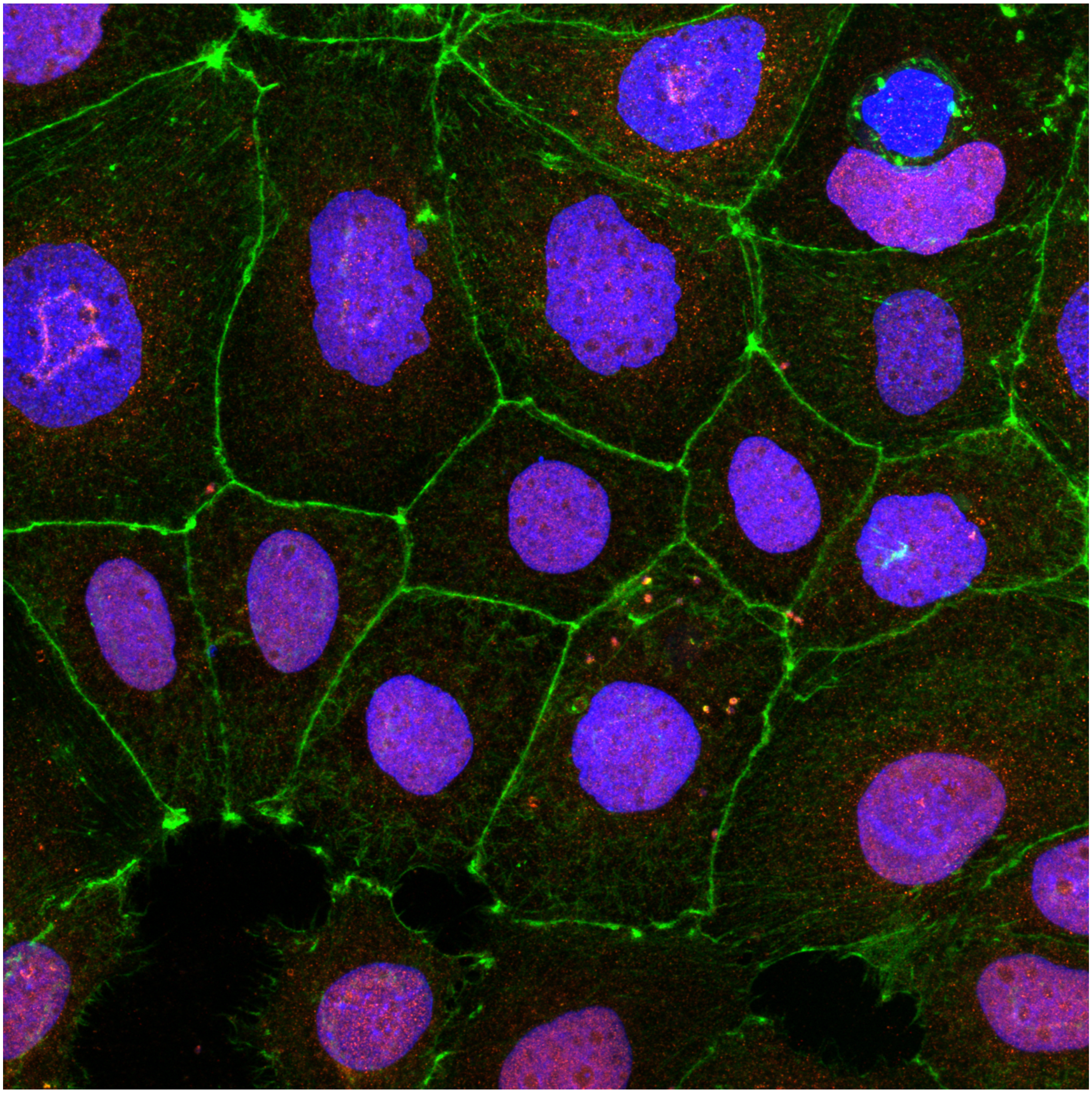

Caco-2/TC7 cells with pH 7 pre-induction Figure 2A - Replicate 1

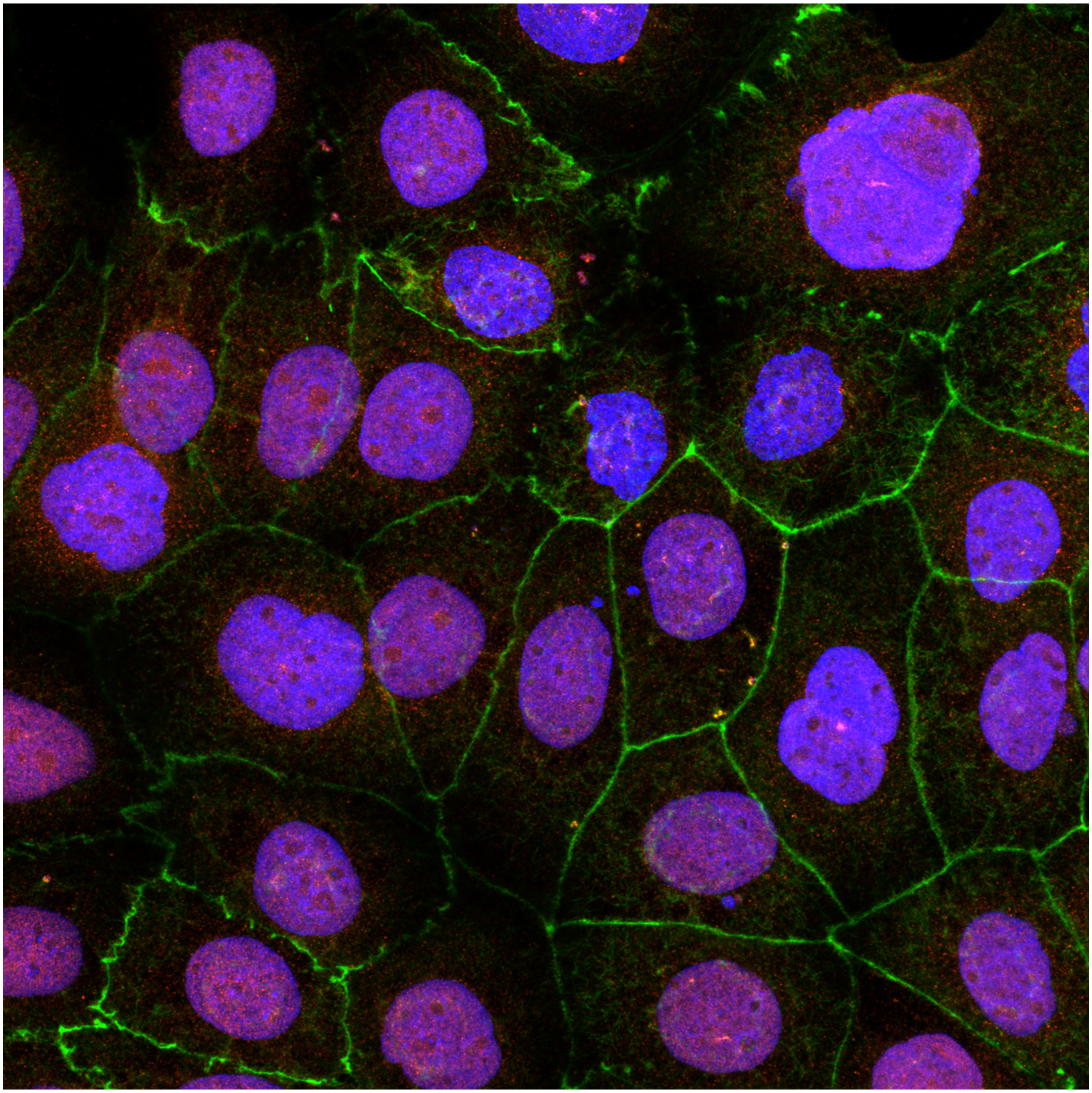

Caco-2/TC7 cells with pH 7 pre-induction Figure 2A - Replicate 2

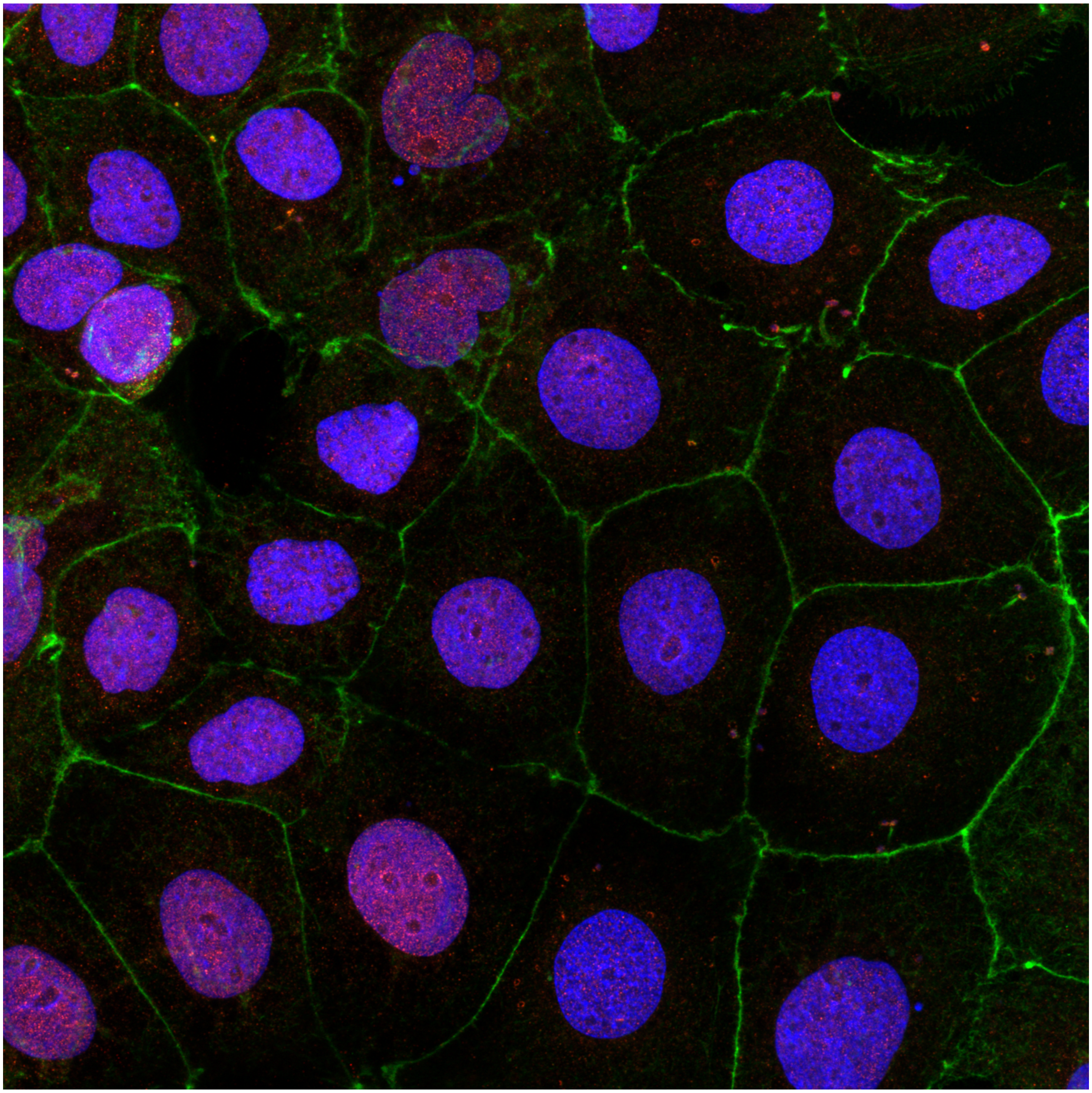

Caco-2/TC7 cells with pH 7 pre-induction Figure 2A - Replicate 3

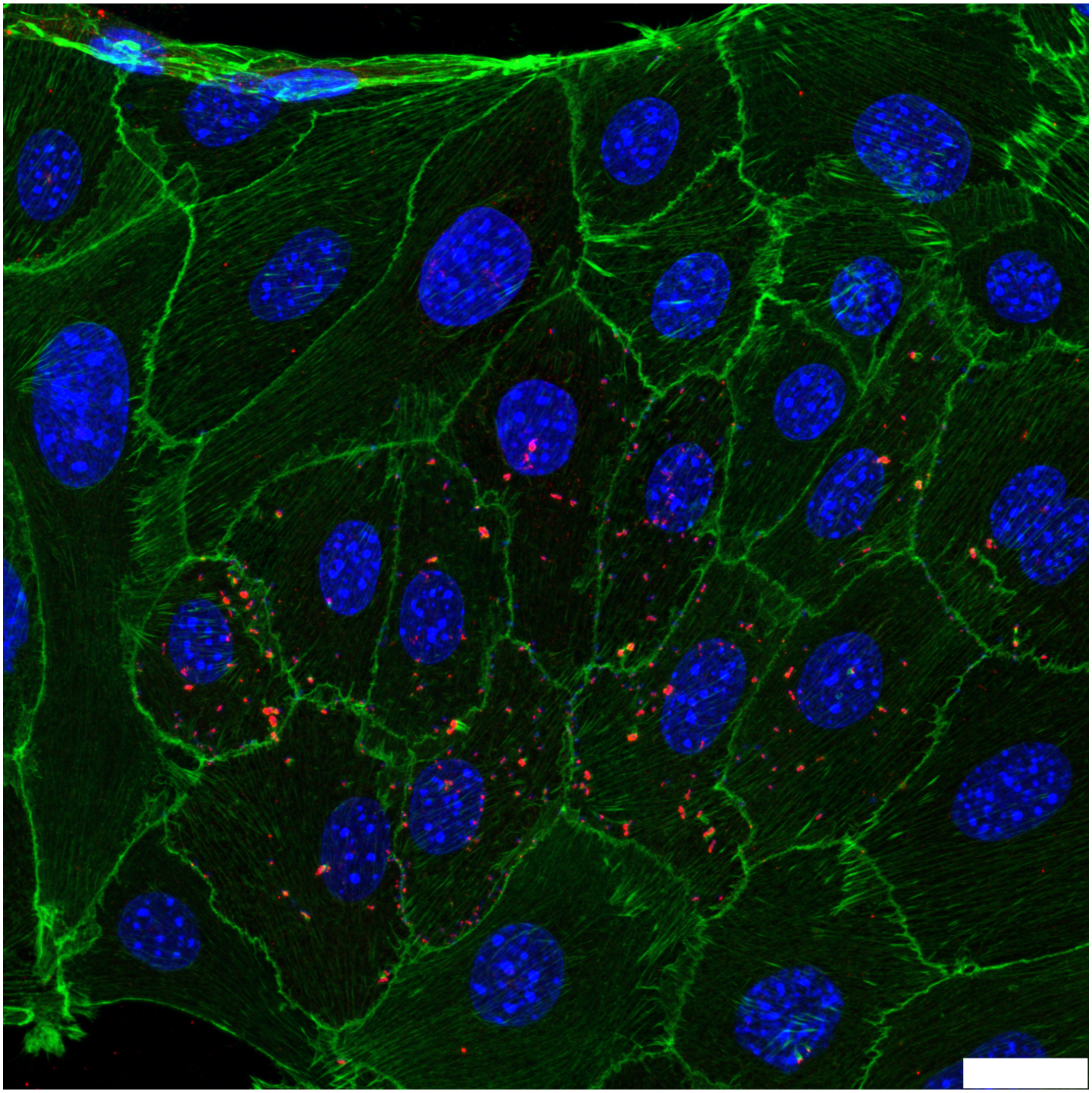

CMT-93 cells with pH 4.8 pre-induction Figure S2I - Replicate 1

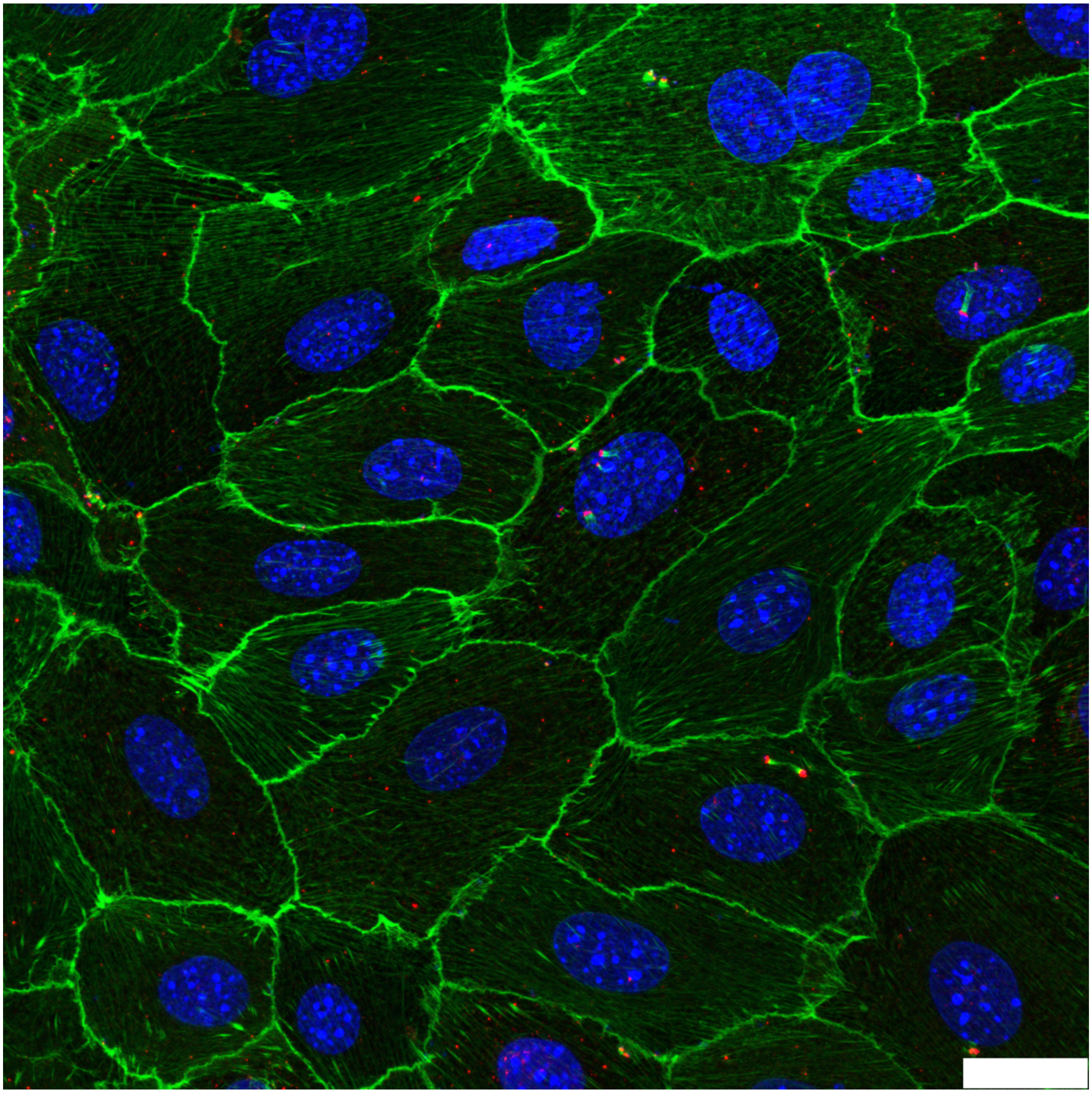

CMT-93 cells with pH 4.8 pre-induction Figure S2I - Replicate 2

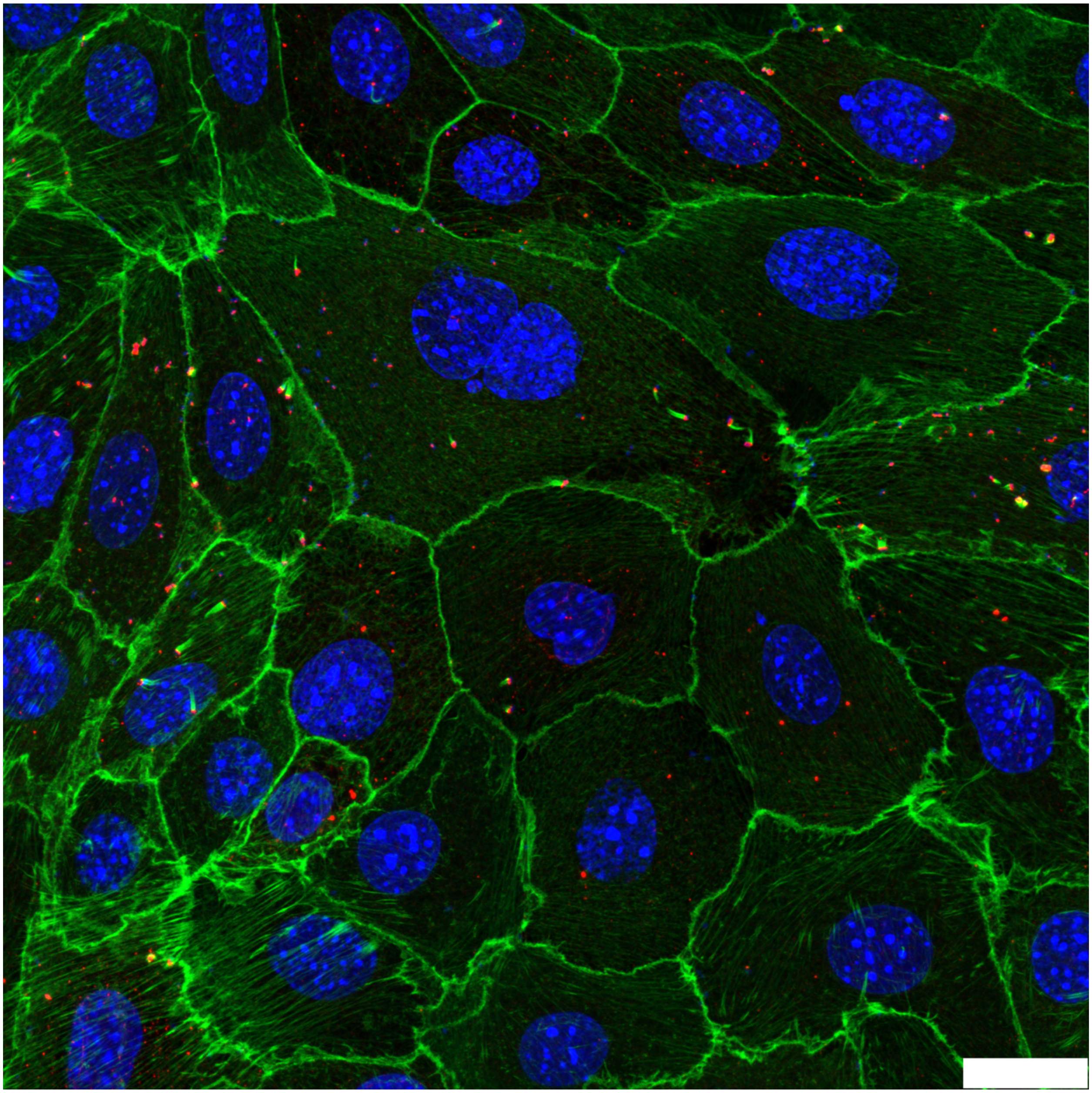

CMT-93 cells with pH 4.8 pre-induction Figure S2I - Replicate 3

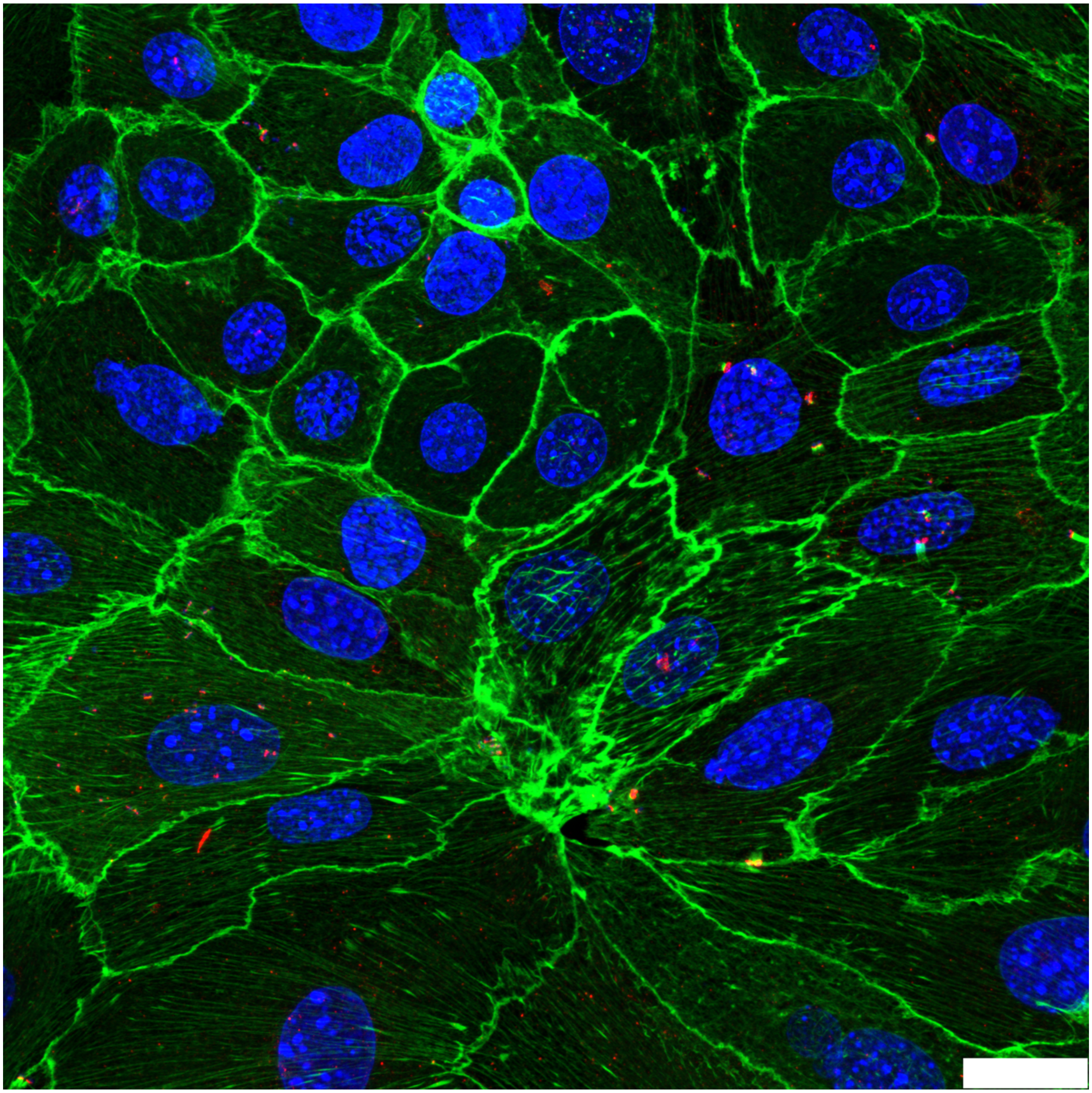

CMT-93 cells with pH 7 pre-induction Figure S2I - Replicate 1

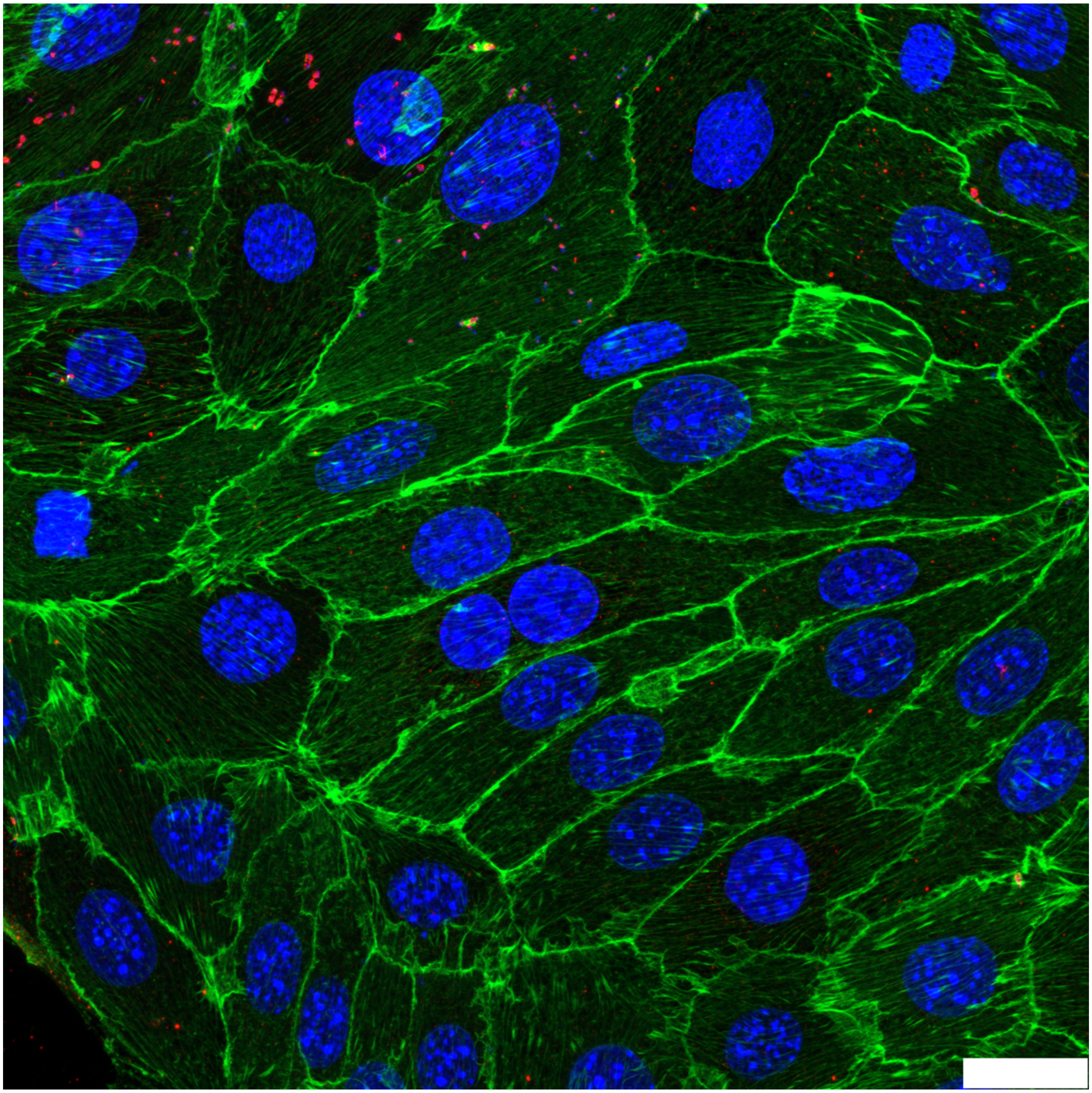

CMT-93 cells with pH 7 pre-induction Figure S2I - Replicate 2

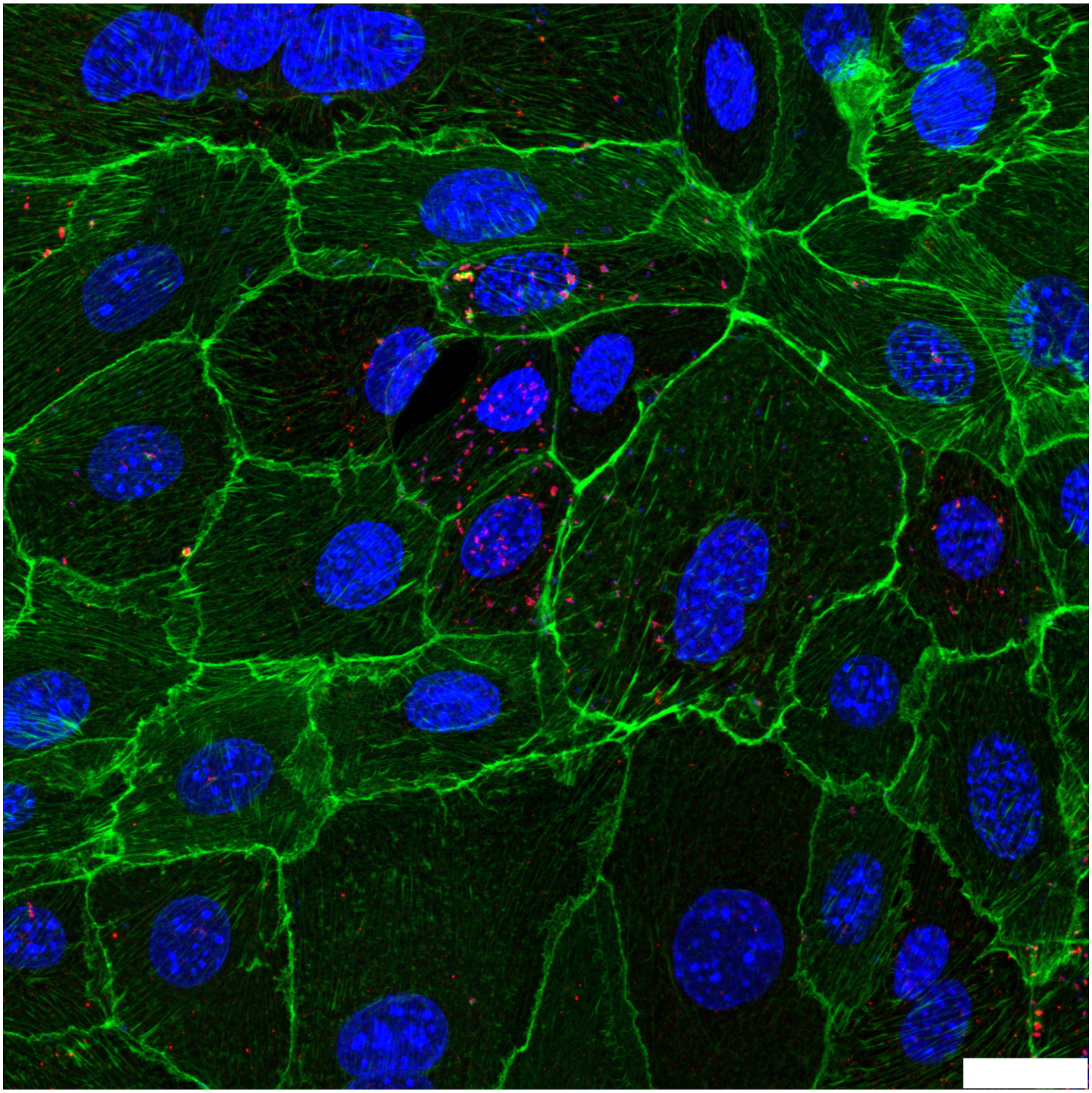

CMT-93 cells with pH 7 pre-induction Figure S2I - Replicate 3

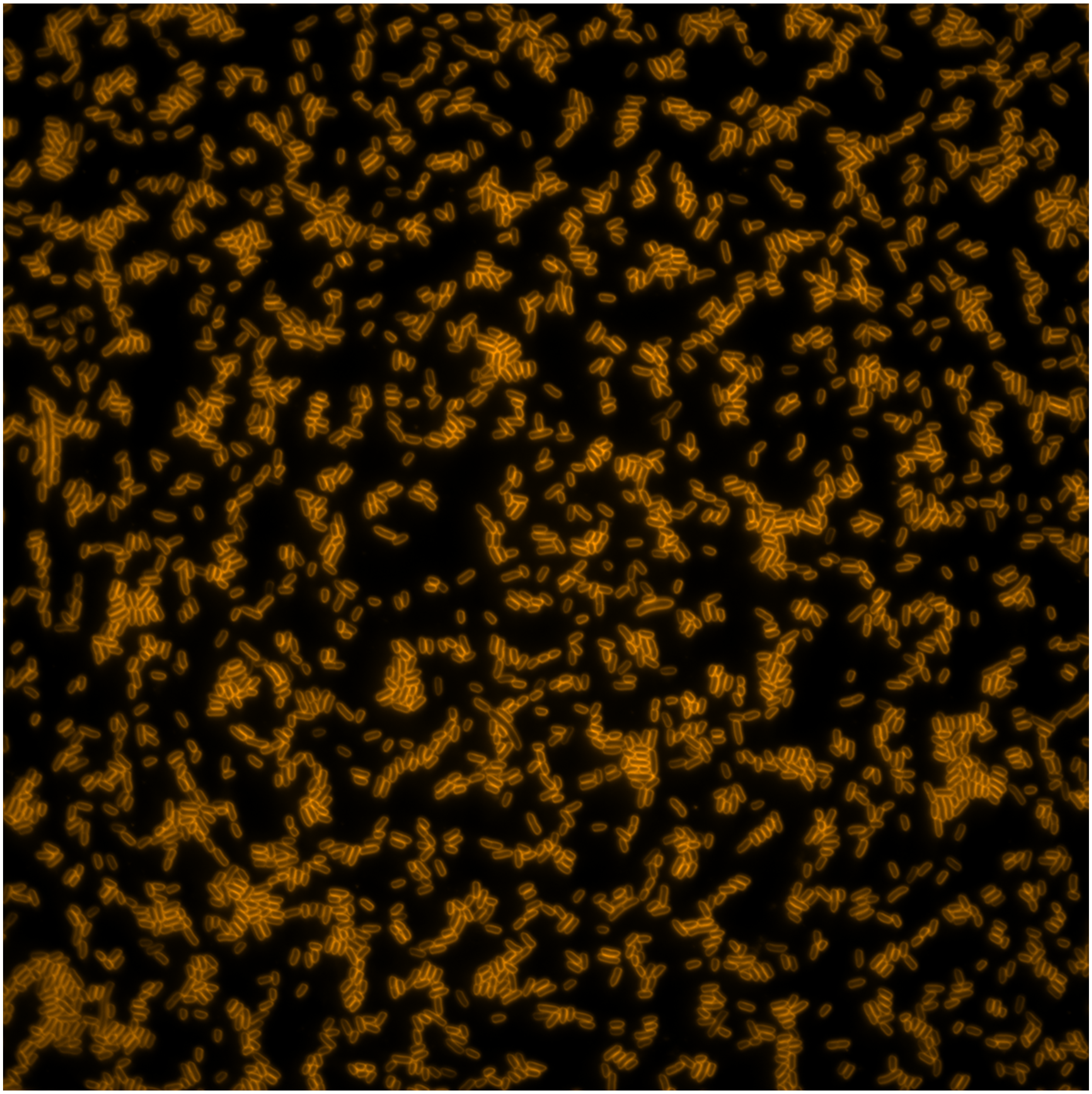

Uncropped image from Figure 4A – pH 7.5

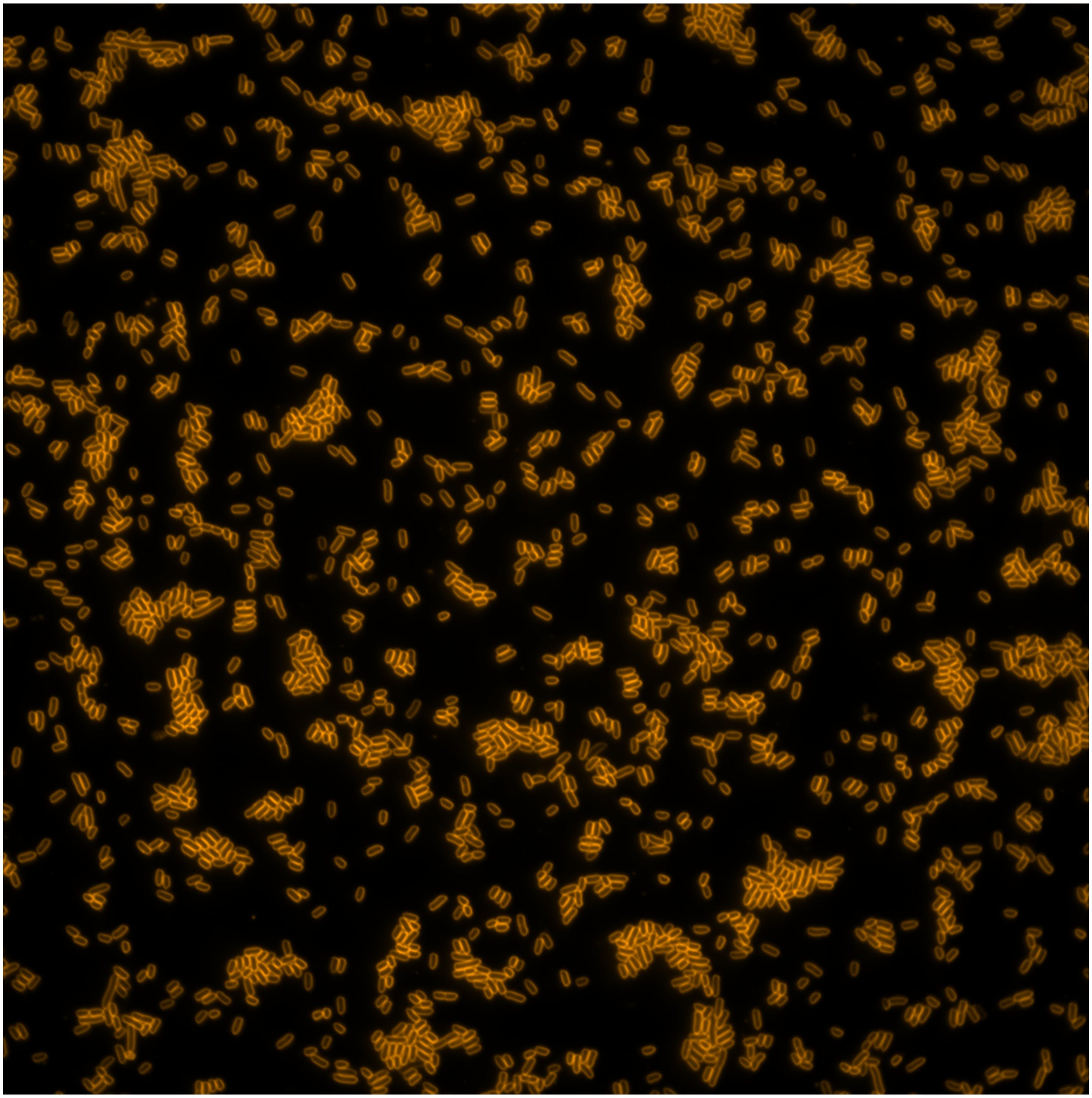

Uncropped image from Figure 4A – pH 7

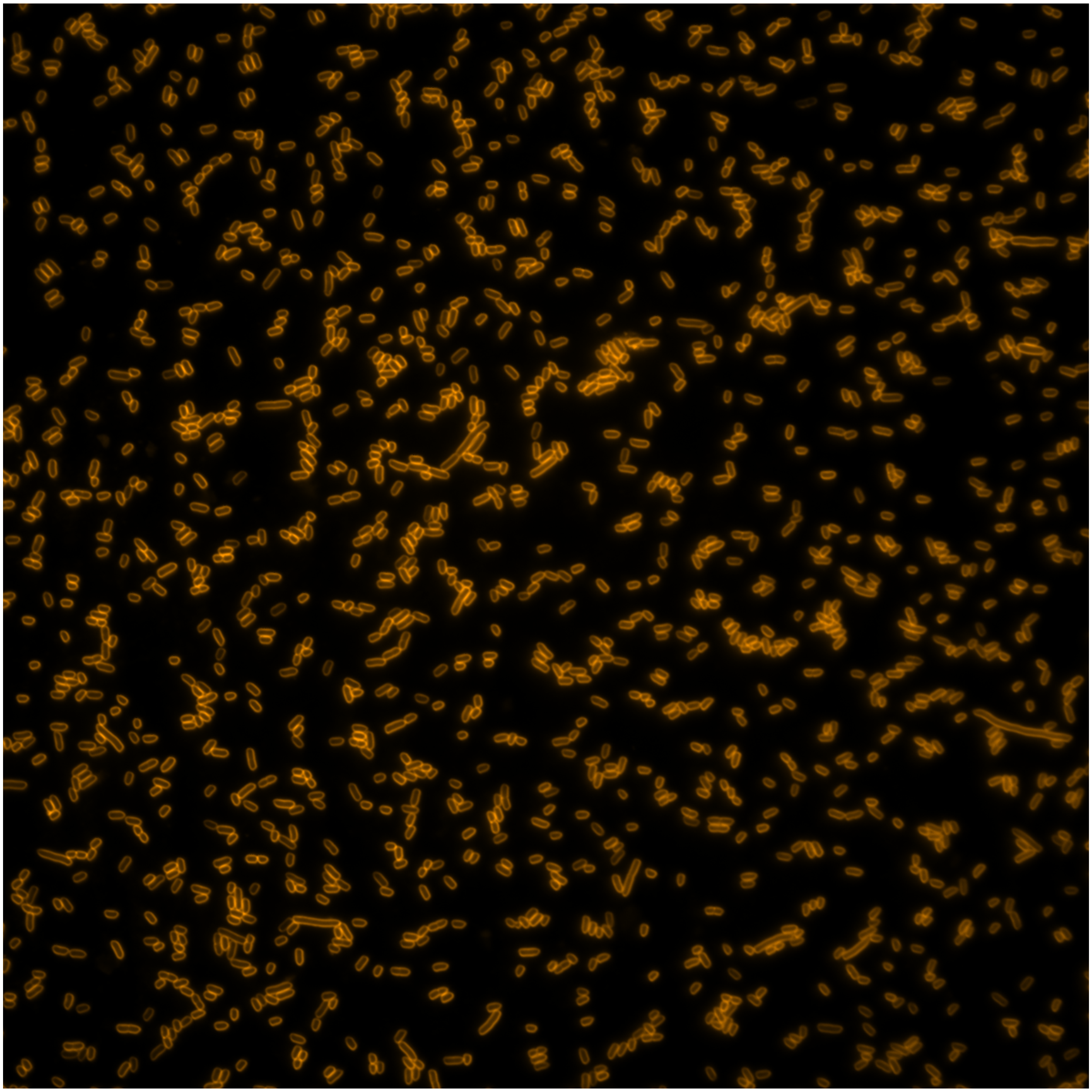
Uncropped image from Figure 4A – pH 6.4

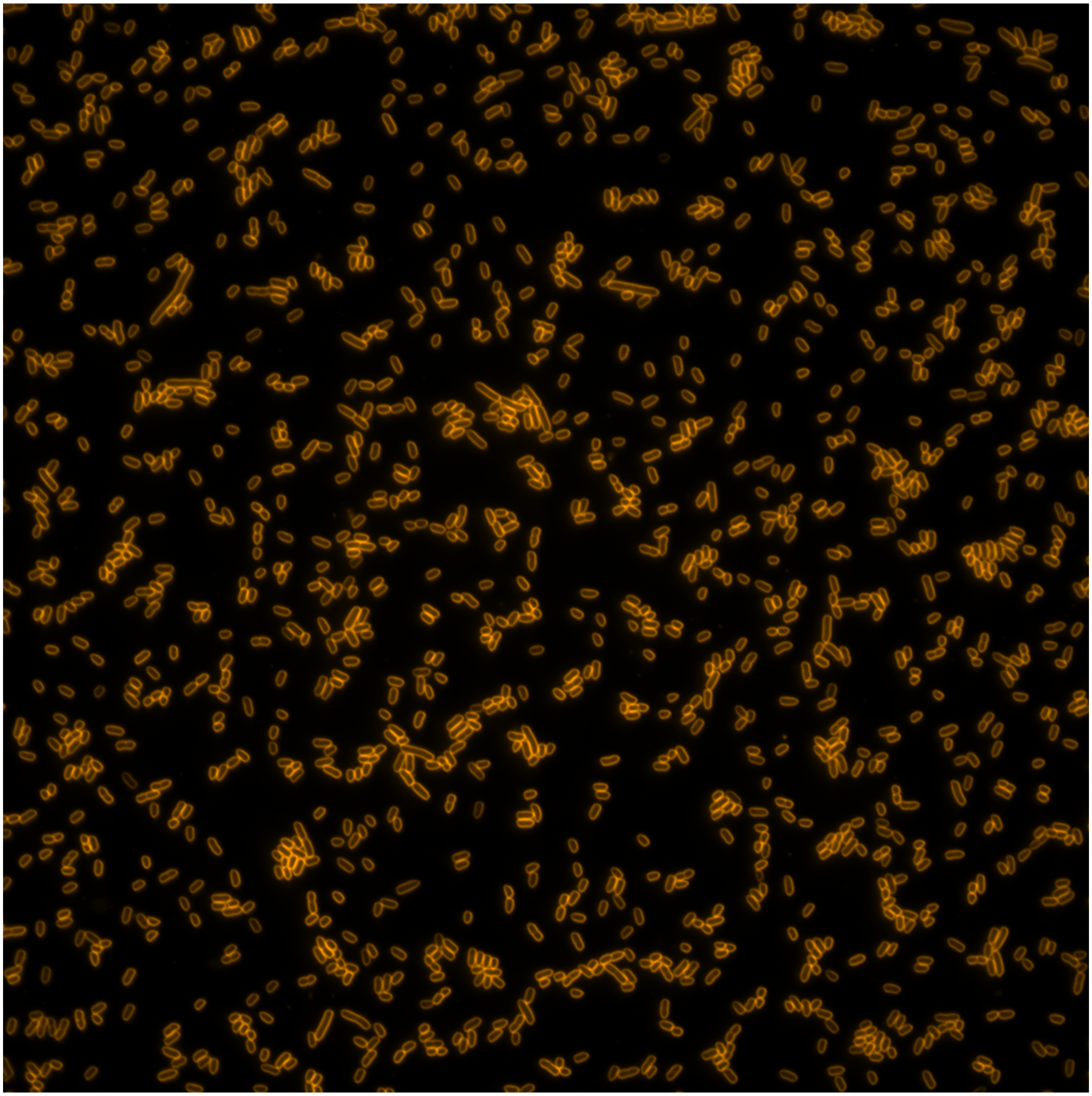
Uncropped image from Figure 4A – pH 5.7

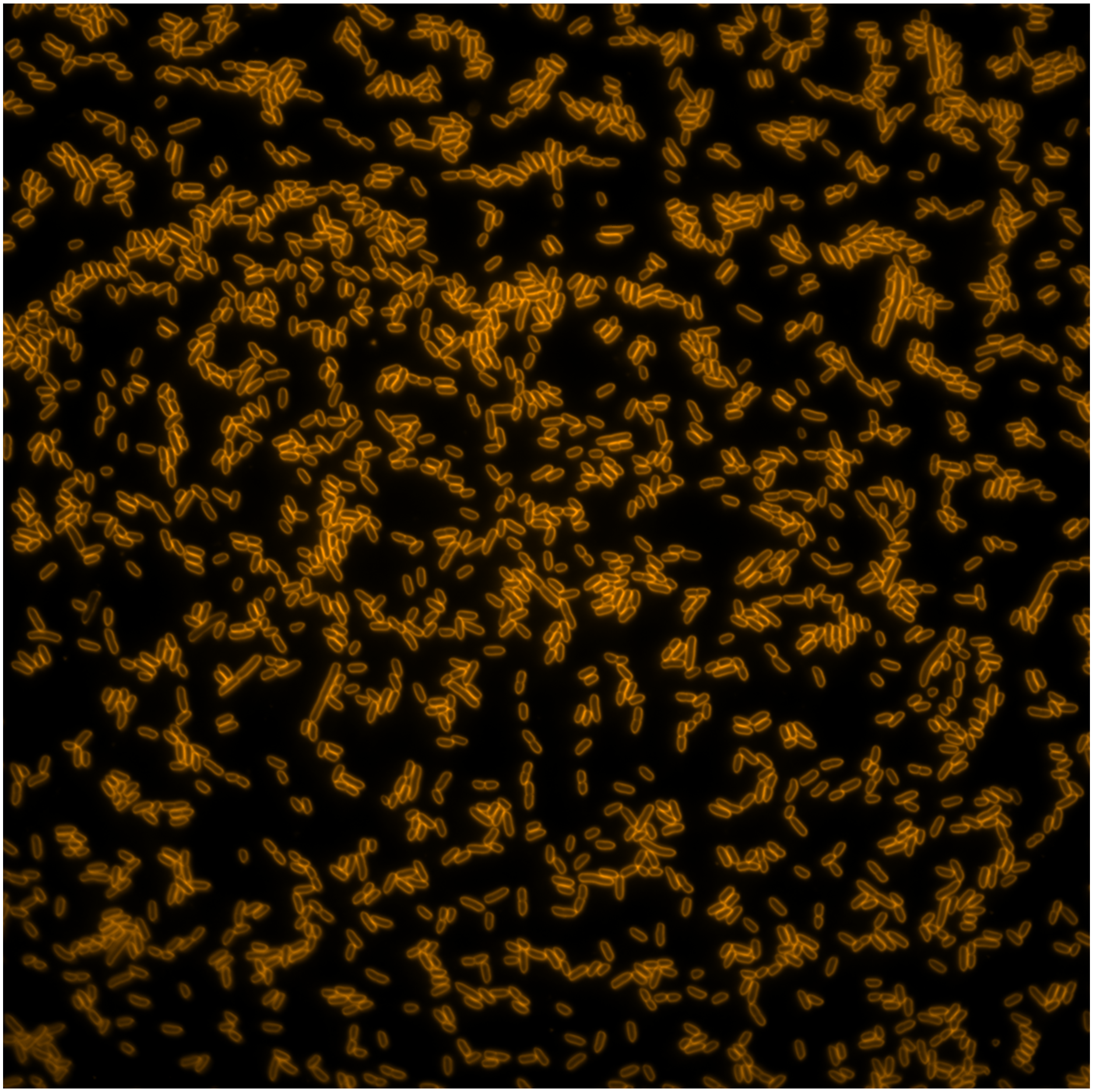

Uncropped image from Figure 4A – pH 4.8

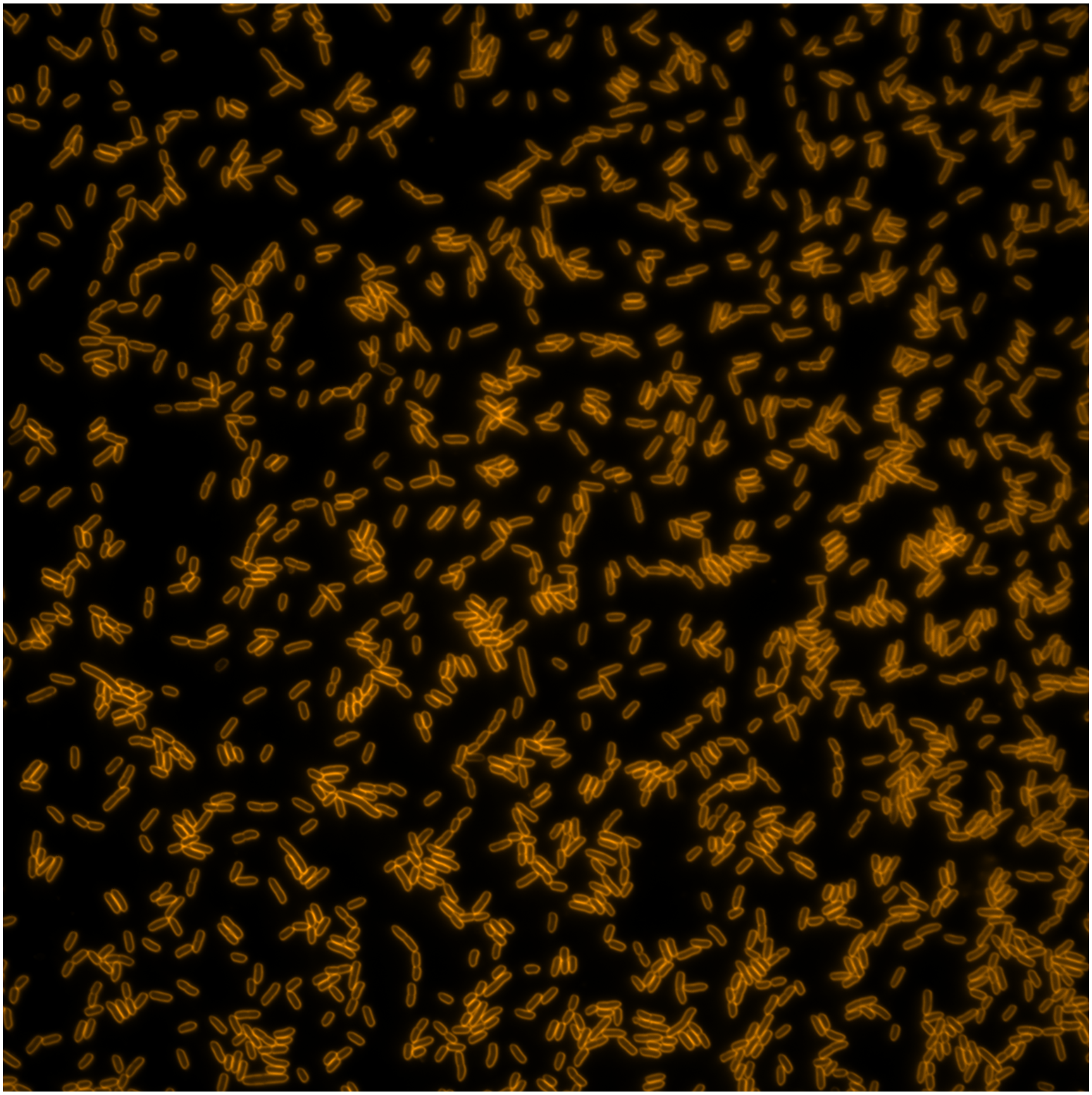

Uncropped image from Figure 4A – pH 4.4

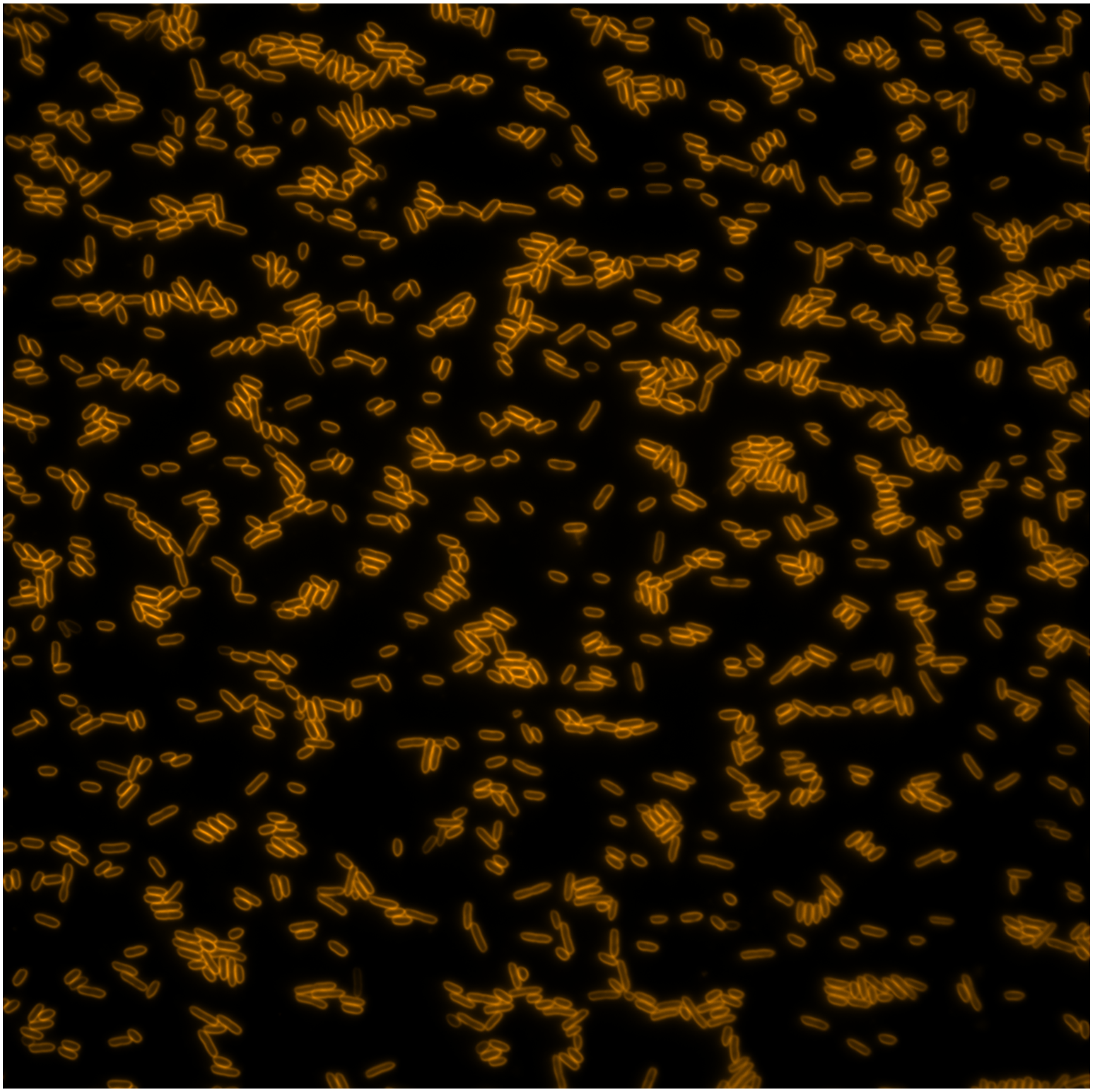

Uncropped image from Figure 4A – pH 4

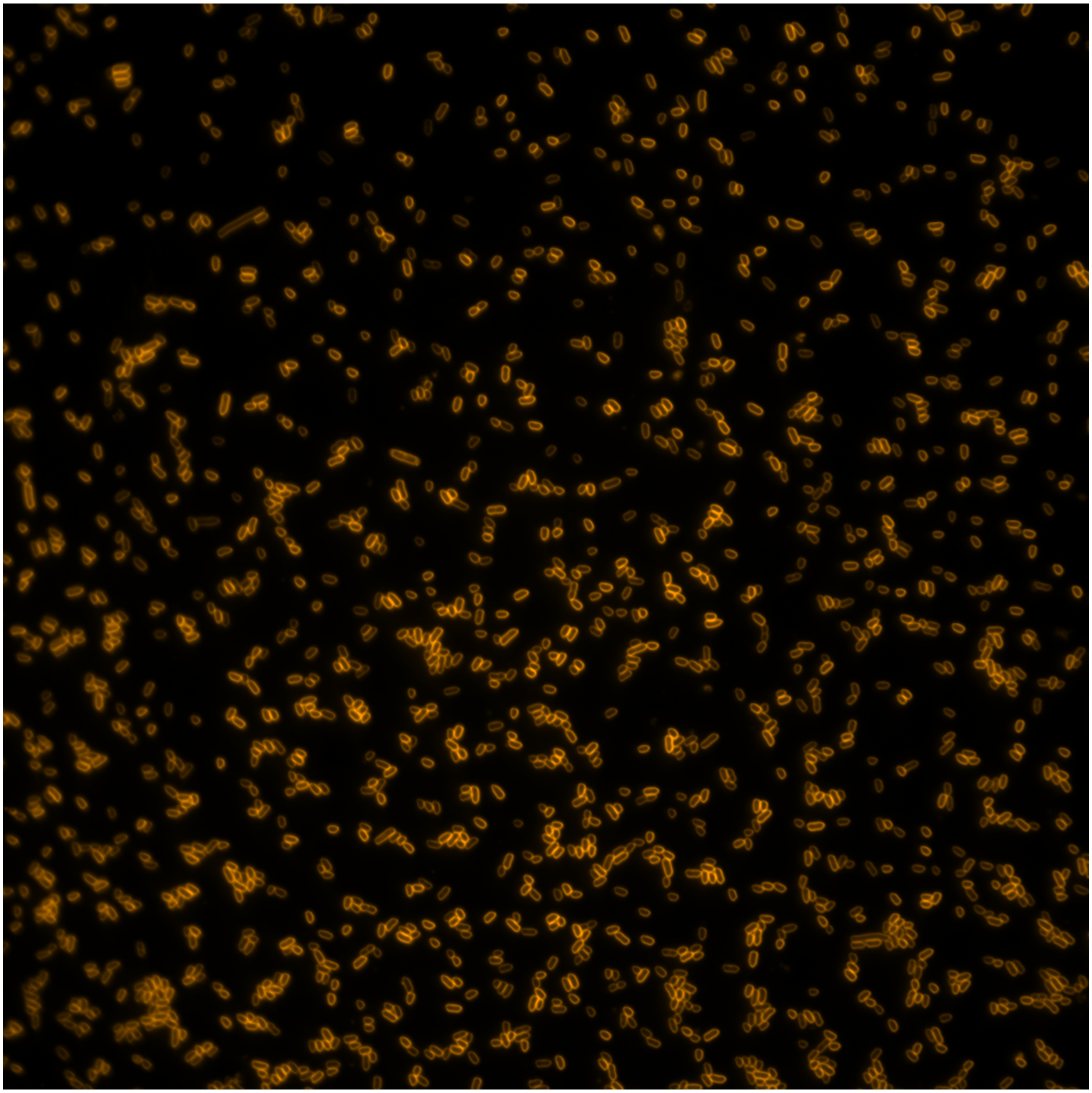

Uncropped image from Figure 4A – pH 3.5

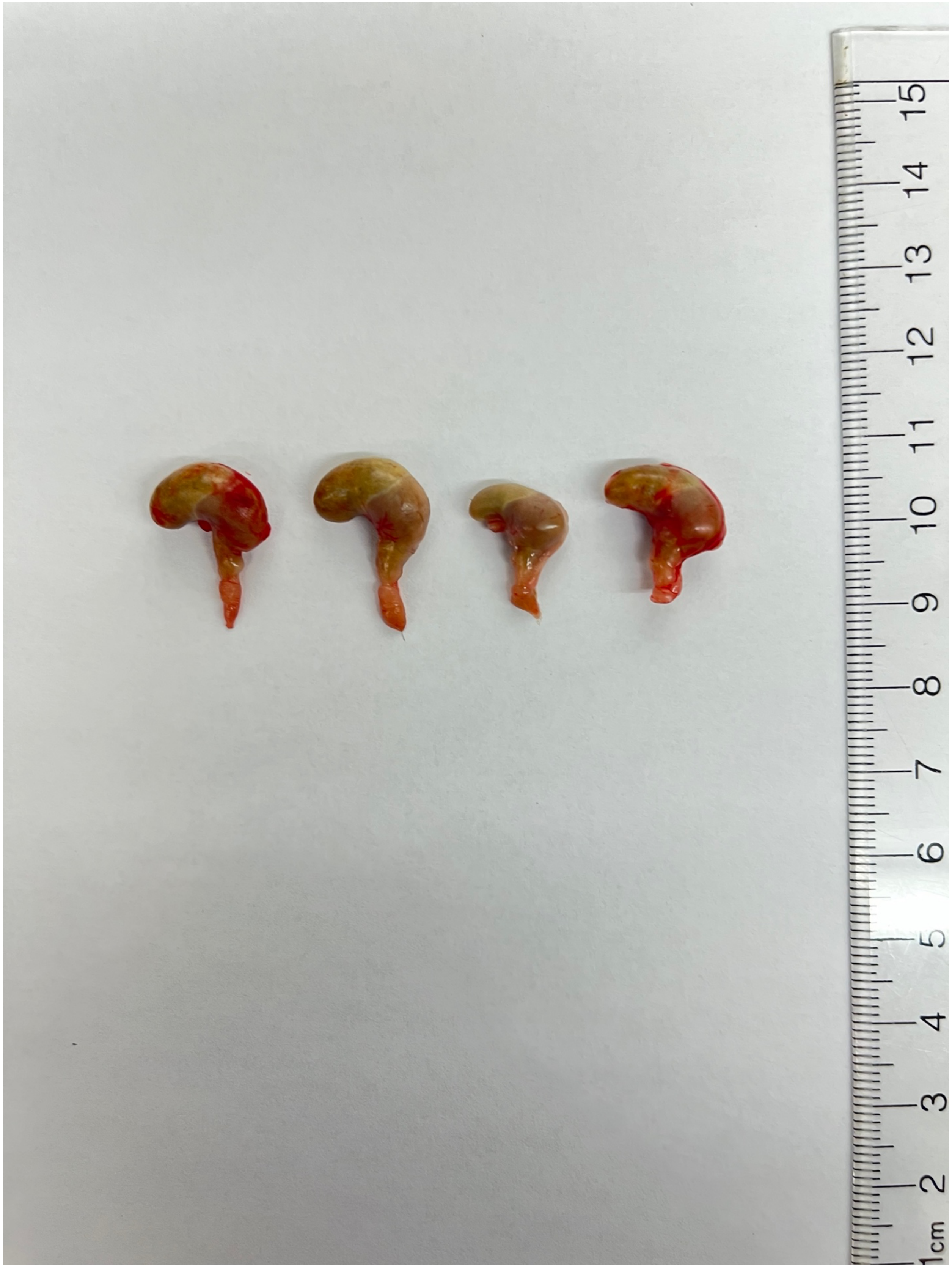

Uncropped image from Figure S5B - Control

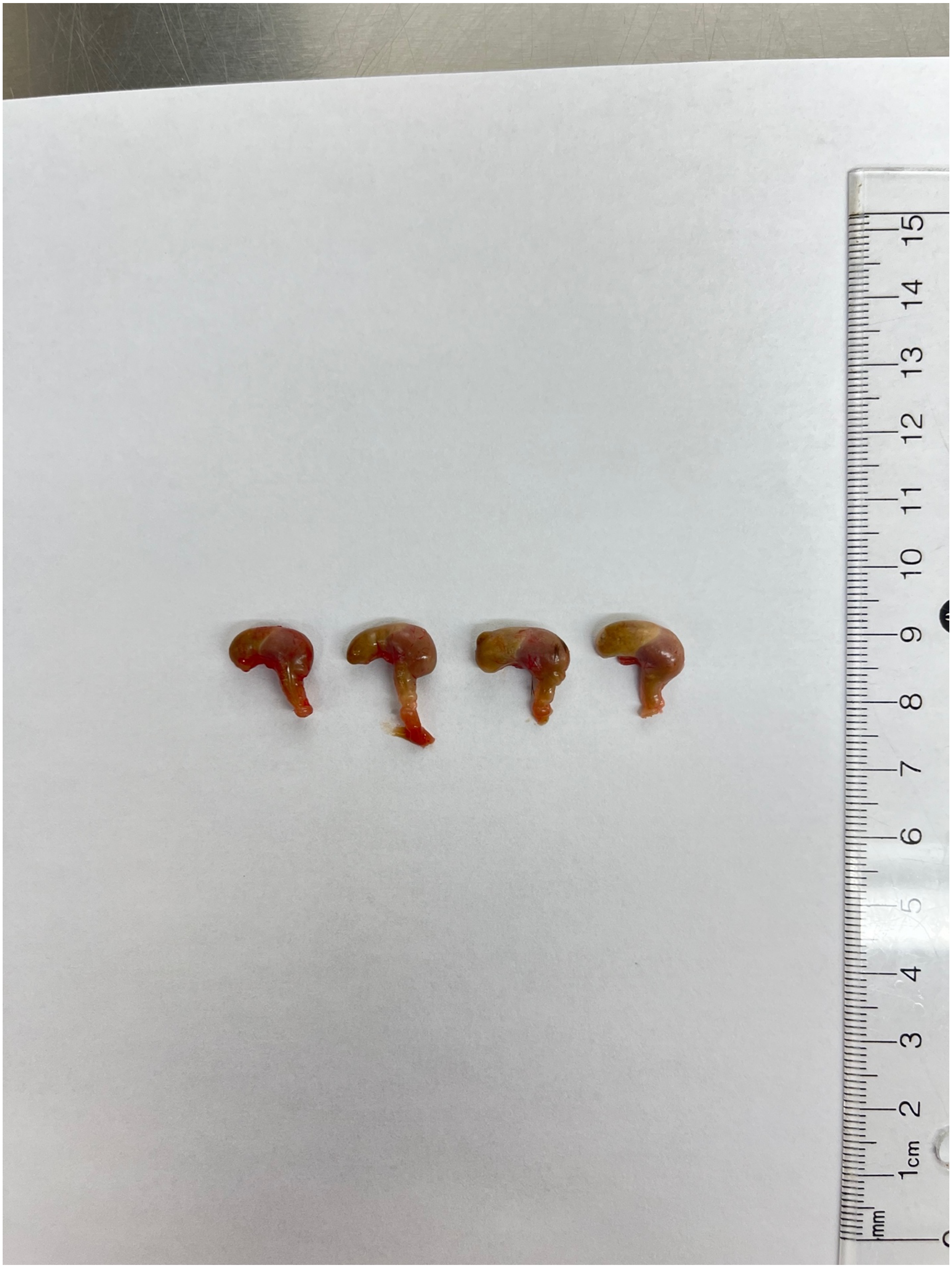

Uncropped image from Figure S5B - Infected
