# Supplementary material for: Both pathogen and host dynamically adapt pH responses along the intestinal tract during enteric bacterial infection
Source: PLoS Biol. 2024 Aug 15;22(8):e3002761. doi: 10.1371/journal.pbio.3002761 (PMC11349234; doi:10.1371/journal.pbio.3002761)
Supplement: S1 Table — (DOCX) [file pbio.3002761.s009.docx]

S1 Table. Bacterial strains used in this study.

| **Strain** | **Description** | **Source or reference** |
| --- | --- | --- |
| DBS100 | Wild-type *C. rodentium*; ATCC 51459 | (Schauer *et al*., 1995) |
| EDL933 | Wild-type EHEC originally isolated from ground beef; ATCC 43895 | (Riley *et al*., 1983) |
| E2348/69 | Wild-type EPEC | (Levine *et al*., 1978) |
| CDC 6516-60 | Wild-type *S.* Typhimurium | ATCC 14028 |
| MFD*pir* | *E. coli* conjugal donor for biparental matings; DAP auxotroph | (Ferrières *et al*., 2010) |
| DBS100 roGFP2 | *C. rodentium* reporter of intra-bacterial redox potential | (van der Heijden *et al*., 2015) |
| DBS100 ∆c*pxRA* | *C. rodentium* lacking genes encoding the CpxRA two-component system | (Thomassin *et al*., 2015) |
| DBS100 *∆escN* | *C. rodentium* unable to produce the type III secretion system | (Deng *et al*., 2004) |
| Mt1B1 | Commensal *E. coli* isolated from murine gut | (Serapio-Palacios *et al*., 2022) |

Table References

1. Riley Lee W., Remis Robert S., Helgerson Steven D., McGee Harry B., Wells Joy G., Davis Betty R., Hebert Richard J., Olcott Ellen S., Johnson Linda M., Hargrett Nancy T., et al. (1983). Hemorrhagic Colitis Associated with a Rare Escherichia coli Serotype. N. Engl. J. Med. *308*, 681–685. https://doi.org/10.1056/NEJM198303243081203.

2. Schauer, D.B., Zabel, B.A., Pedraza, I.F., O’Hara, C.M., Steigerwalt, A.G., and Brenner, D.J. (1995). Genetic and biochemical characterization of Citrobacter rodentium sp. nov. J. Clin. Microbiol. *33*, 2064–2068. https://doi.org/10.1128/jcm.33.8.2064-2068.1995.

3. Levine, M.M., Bergquist, E.J., Nalin, D.R., Waterman, D.H., Hornick, R.B., Young, C.R., and Sotman, S. (1978). Escherichia coli strains that cause diarrhoea but do not produce heat-labile or heat-stable enterotoxins and are non-invasive. Lancet Lond. Engl. *1*, 1119–1122. https://doi.org/10.1016/s0140-6736(78)90299-4.

4. Ferrières, L., Hémery, G., Nham, T., Guérout, A.-M., Mazel, D., Beloin, C., and Ghigo, J.-M. (2010). Silent mischief: bacteriophage Mu insertions contaminate products of Escherichia coli random mutagenesis performed using suicidal transposon delivery plasmids mobilized by broad-host-range RP4 conjugative machinery. J. Bacteriol. *192*, 6418–6427. https://doi.org/10.1128/JB.00621-10.

5. van der Heijden, J., and Finlay, B.B. (2015). In vitro Real-time Measurement of the Intra-bacterial Redox Potential. Bio-Protoc. *5*, 1–9. https://doi.org/10.21769/bioprotoc.1579.

6. Thomassin, J.-L., Giannakopoulou, N., Zhu, L., Gross, J., Salmon, K., Leclerc, J.-M., Daigle, F., Le Moual, H., and Gruenheid, S. (2015). The CpxRA Two-Component System Is Essential for Citrobacter rodentium Virulence. Infect. Immun. *83*, 1919–1928. https://doi.org/10.1128/IAI.00194-15.

7. Deng, W., Puente, J.L., Gruenheid, S., Li, Y., Vallance, B.A., Vázquez, A., Barba, J., Ibarra, J.A., O’Donnell, P., Metalnikov, P., et al. (2004). Dissecting virulence: systematic and functional analyses of a pathogenicity island. Proc. Natl. Acad. Sci. U. S. A. *101*, 3597–3602. https://doi.org/10.1073/pnas.0400326101.

8. Serapio-Palacios, A., Woodward, S.E., Vogt, S.L., Deng, W., Creus-Cuadros, A., Huus, K.E., Cirstea, M., Gerrie, M., Barcik, W., Yu, H., et al. (2022). Type VI secretion systems of pathogenic and commensal bacteria mediate niche occupancy in the gut. Cell Rep. *39*. https://doi.org/10.1016/j.celrep.2022.110731.
