# Supplementary material for: Both pathogen and host dynamically adapt pH responses along the intestinal tract during enteric bacterial infection
Source: PLoS Biol. 2024 Aug 15;22(8):e3002761. doi: 10.1371/journal.pbio.3002761 (PMC11349234; doi:10.1371/journal.pbio.3002761)
Supplement: S2 Table — (DOCX) [file pbio.3002761.s010.docx]

S2 Table. Oligonucleotide primers and plasmids used for making deletion constructs of *C. rodentium ldcC*.

| *Primers* | | |
| --- | --- | --- |
| **Primer Name** | **Sequence** | **Purpose** |
| dldcC1.F | TGAATTCCCGGGAGAGCTCCCGCATCACCTATTACAAAGAGTTCGCG | *ldcC* deletion fragment 1 forward |
| dldcC1.R | TCATATGTTTTTTAAAATGATATTCATGCGTTCCTCCTGGAAAAACC | *ldcC* deletion fragment 1 reverse |
| dldcC2.F | CGCATGAATATCATTTTAAAAAACATATGACGGCTTGCCTTTATGCG | *ldcC* deletion fragment 2 forward |
| dldcC2.R | GCCCGATCCCAAGCTTCTTCTAGAGCGGACAGCCCTGCCGGAC | *ldcC* deletion fragment 2 reverse |
| Check_ldcC_F | CACTTATCCTGCGTGGAACA | Verifying *ldcC* deletion |
| Check_ldcC_R | GGGCGATAGCCTGTTCAAT | Verifying *ldcC* deletion |
| *Plasmids* | | |
| **Plasmid** | **Description** | **Reference** |
| pRE112 | Suicide vector for allelic exchange; Cam^R^ | (Edwards *et al*., 1998) |

Table References

1. Edwards, R.A., Keller, L.H., and Schifferli, D.M. (1998). Improved allelic exchange vectors and their use to analyze 987P fimbria gene expression. Gene *207*, 149–157. https://doi.org/10.1016/s0378-1119(97)00619-7.
