# Supplementary material for: Both pathogen and host dynamically adapt pH responses along the intestinal tract during enteric bacterial infection
Source: PLoS Biol. 2024 Aug 15;22(8):e3002761. doi: 10.1371/journal.pbio.3002761 (PMC11349234; doi:10.1371/journal.pbio.3002761)
Supplement: S3 Table — (DOCX) [file pbio.3002761.s011.docx]

S3 Table. Oligonucleotide primers used for RT-qPCR.

| **Primer Name** | **Sequence** | **Gene Target** |
| --- | --- | --- |
| *Bacterial gene expression* | | |
| dnaQ_F | GTCAGGCCCGCAAATTAC | *dnaQ*, endogenous control |
| dnaQ_R | TCTCCACCAGATCGAGACG | *dnaQ*, endogenous control |
| regA_F | TGCGGGATGCTCAACTTCTT | *regA* |
| regA_R | TTTTACGCCGCGTGTGATTG | *regA* |
| ler_F | CGGAGCTCATCAAAAGGGGT | *ler* |
| ler_R | GTTGTCGACCCACTCCAGAC | *ler* |
| eae_F | GGTTAATCTGCAGAGCGGTAA | *eae* |
| eae_R | GAACGGTAATAAGAAGTCCAGTGAA | *eae* |
| tir_F | TTGCATCGACCCAATGGTCA | *tir* |
| tir_R | AGTGCTTTGGATACCCTGCC | *tir* |
| nleB1_F | GCTCCTCTCCAGACTGTTTCC | *nleB1* |
| nleB1_R | GCTCTGGCCTTGCTTCAAAC | *nleB1* |
| espA_F | AATCACCGGCGCTTAACTCA | *espA* |
| espA_R | TTCACGCACAAAGCGAACTG | *espA* |
| fimA_F | TATGTCTGCCCTGTCCCTGA | *fimA* |
| fimA_R | TTAAAATGCACTGTGCCGCC | *fmA* |
| kfcC_F | TGCAACCGAGACGGATAAGG | *kfcC* |
| kfcC_R | TTGCCGGGCGTTTTAATTGG | *kfcC* |
| degP_F | CATTAACACCGCGATCCTC | *degP* |
| degP_R | CCATATTGCTCGGGATAGCA | *degP* |
| cpxA_F | GGTGTTGATGTTGCCCAAGC | *cpxA* |
| cpxA_R | GCCTCGACGTGTTGCTCTAT | *cpxA* |
| rpoS_F | GAAGACACCACGCAGGATGA | *rpoS* |
| rpoS_R | CCGCTTCATAACCCAGCAGA | *rpoS* |
| recA_F | TGCCACTACCTGGCTGAAAG | *recA* |
| recA_R | CGTGGAGTCCTGGTTGTTGA | *recA* |
| fur_F | ACCATGTTTCGCCGCAATCT | *fur* |
| fur_R | TGAACCAGTTCGATGACGCC | *fur* |
| asr_F | GGCTTGCCGCTTTGTGATG | *asr* |
| asr_R | TAGCTCTGGTTGTTGCCGCT | *asr* |
| yodD_F | CCGCAAAACGTGAAGTCAGC | *yodD* |
| yodD_R | GCTTCCGCTACGATGAACCT | *yodD* |
| atpD_F | ACCACGAAATGACCGACTCC | *atpD* |
| atpD_R | AGAACGTCACGACCTTCGTC | *atpD* |
| ldcC_F | AGCTACCCCATTGTGGCTTC | *ldcC* |
| ldcC_R | CTGTCAGACTCTTCCCGCAG | *ldcC* |
| *Host gene expression* | | |
| b2m_F | CCAAGACCGTCTACTGGGGT | *B2M*, endogenous control |
| b2m_R | TCTGCTGGCACCACAGATCA | *B2M*, endogenous control |
| tjp1_F | CCCTGAAAGAAGCGATTCAG | *TJP1* |
| tjp1_R | CCCGCCTTCTGTATCTGTGT | *TJP1* |
| atp4b_F | GACGTGTATGGGGAAAGAGGG | *ATP4B* |
| atp4b_R | GATGCTGGGGTATAGCCTGCTAAG | *ATP4B* |
| hrh2_1_F | AAGCCCAGTCAGGACACGAC | *HRH2* |
| hrh2_1_R | TCCCAGCTTCCAGATGAAGGA | *HRH2* |
| hdc_F | CTGGACAGAAGCAGAGGAACC | *HDC* |
| hdc_R | GCAGGTTTTCCCCTATGCTCA | *HDC* |
| gast_F | GAGATGGGGTCCCTTGCTTG | *GAST* |
| gast_R | CGGGAATCACGGACAACAGT | *GAST* |
| chrm3_F | CAAGCGGAAAAGGATGTCGC | *CHRM3* |
| chrm3_R | GATGTTGTAGGGGGTCCACG | *CHRM3* |
| pac1_F | CCTCTGACCTACTGCCACCC | *PAC1* |
| pac1_R | AGAGGACATCTGCTCTGGTACT | *PAC1* |
| cck2r_F | CTAGGGCTCCGCTTTGATGG | *CCK2R* |
| cck2r_R | CCTCGTGTTTTTCACCCACCT | *CCK2R* |
| vip_F | ACCGTCTACGCAGAGGTACT | *VIP* |
| vip_R | GCATTCACTGAGGTGGGCT | *VIP* |
| vpac2_F | AGTATGGGAGGTGCTATGGGT | *VPAC2* |
| vpac2_R | GCTGGGCAGAGGTTGATGAT | *VPAC2* |
| sst_F | ACCAGTGCTTTTACGCACCA | *SST* |
| sst_R | CCACTTTAGATCGTGTCGCCT | *SST* |
| vipr1_F | GATAAGCACCCCCATTGTGCT | *VPAC1* |
| vipr1_R | CTCGCCATTGAGGAAGCAGTA | *VPAC1* |
