# Supplementary material for: Both pathogen and host dynamically adapt pH responses along the intestinal tract during enteric bacterial infection
Source: PLoS Biol. 2024 Aug 15;22(8):e3002761. doi: 10.1371/journal.pbio.3002761 (PMC11349234; doi:10.1371/journal.pbio.3002761)
Supplement: Attachment — Submitted filename: PLOS Biology Response to Reviewers_Final.docx [file pbio.3002761.s012.docx]

Dear Dr Finlay,

I would like to apologize for the duration of the revision process. Thank you for your patience while your manuscript "Pathogen and host adapt pH responses during enteric infection" was peer-reviewed at PLOS Biology. It has now been evaluated by the PLOS Biology editors, an Academic Editor with relevant expertise, and by several independent reviewers. 

In light of the reviews, which you will find at the end of this email, we would like to invite you to revise the work to thoroughly address the reviewers' reports. As you will see below, specifically Reviewer #2 and 3 have concerns regarding the lack of support of some conclusions. Reviewer #2 suggests additional experiments such as testing infection outcomes in LB and following a transcriptomics approach, as well as additional in vivo experiments. Reviewer #3 asks to demonstrate the reduced acid tolerance using an LdcC mutant and to quantify bacterial burden across groups.

IMPORTANT: although Reviewer #2’s suggestions regarding additional transcriptomic analysis and in vivo experiments could potentially strengthen your study, after discussion with the Academic Editor and the reviewers, we determined to leave to your discretion to follow such suggestions. Additionally, the Academic Editor has provided additional requests regarding presentation and clarification of some points in the text and choice of experiments (see the foot of this email). Therefore, with the exception of additional in vivo experiments, addressing the concerns raised by all reviewers and the Academic Editor is necessary for your manuscript to be considered for publication in PLOS Biology.

Given the extent of revision needed, we cannot make a decision about publication until we have seen the revised manuscript and your response to the reviewers' comments. Your revised manuscript is likely to be sent for further evaluation by all or a subset of the reviewers.

We expect to receive your revised manuscript within 3 months. Please email us ([plosbiology@plos.org](mailto:plosbiology@plos.org)) if you have any questions or concerns, or would like to request an extension. 

At this stage, your manuscript remains formally under active consideration at our journal; please notify us by email if you do not intend to submit a revision so that we may withdraw it.

**IMPORTANT - SUBMITTING YOUR REVISION**

Your revisions should address the specific points made by each reviewer. Please submit the following files along with your revised manuscript:

1. A 'Response to Reviewers' file - this should detail your responses to the editorial requests, present a point-by-point response to all of the reviewers' comments, and indicate the changes made to the manuscript. 
*NOTE: In your point-by-point response to the reviewers, please provide the full context of each review. Do not selectively quote paragraphs or sentences to reply to. The entire set of reviewer comments should be present in full and each specific point should be responded to individually, point by point.
You should also cite any additional relevant literature that has been published since the original submission and mention any additional citations in your response. 

2. In addition to a clean copy of the manuscript, please also upload a 'track-changes' version of your manuscript that specifies the edits made. This should be uploaded as a "Revised Article with Changes Highlighted" file type. 

*Re-submission Checklist*
When you are ready to resubmit your revised manuscript, please refer to this re-submission checklist: <https://plos.io/Biology_Checklist>

To submit a revised version of your manuscript, please go to <https://www.editorialmanager.com/pbiology/> and log in as an Author. Click the link labelled 'Submissions Needing Revision' where you will find your submission record. 

Please make sure to read the following important policies and guidelines while preparing your revision:

*Published Peer Review*
Please note while forming your response, if your article is accepted, you may have the opportunity to make the peer review history publicly available. The record will include editor decision letters (with reviews) and your responses to reviewer comments. If eligible, we will contact you to opt in or out. Please see here for more details:
<https://blogs.plos.org/plos/2019/05/plos-journals-now-open-for-published-peer-review/>

*PLOS Data Policy*
Please note that as a condition of publication PLOS' data policy (<http://journals.plos.org/plosbiology/s/data-availability>) requires that you make available all data used to draw the conclusions arrived at in your manuscript. If you have not already done so, you must include any data used in your manuscript either in appropriate repositories, within the body of the manuscript, or as supporting information (N.B. this includes any numerical values that were used to generate graphs, histograms etc.). For an example see here: <http://www.plosbiology.org/article/info%3Adoi%2F10.1371%2Fjournal.pbio.1001908#s5> 

*Blot and Gel Data Policy*
We require the original, uncropped and minimally adjusted images supporting all blot and gel results reported in an article's figures or Supporting Information files. We will require these files before a manuscript can be accepted so please prepare them now, if you have not already uploaded them. Please carefully read our guidelines for how to prepare and upload this data: <https://journals.plos.org/plosbiology/s/figures#loc-blot-and-gel-reporting-requirements> 

*Protocols deposition*
To enhance the reproducibility of your results, we recommend that if applicable you deposit your laboratory protocols in [protocols.io](http://protocols.io/), where a protocol can be assigned its own identifier (DOI) such that it can be cited independently in the future. Additionally, PLOS ONE offers an option for publishing peer-reviewed Lab Protocol articles, which describe protocols hosted on [protocols.io](http://protocols.io/). Read more information on sharing protocols at <https://plos.org/protocols?utm_medium=editorial-email&utm_source=authorletters&utm_campaign=protocols>

Thank you again for your submission to our journal. We hope that our editorial process has been constructive thus far, and we welcome your feedback at any time. Please don't hesitate to contact us if you have any questions or comments.

Sincerely,
Melissa


Melissa Vazquez Hernandez, Ph.D.
Associate Editor
PLOS Biology
[mvazquezhernandez@plos.org](mailto:mvazquezhernandez@plos.org)

*We thank the editor for their thoughtful consideration of the requested revisions and hope you will now find the resubmitted manuscript appropriate for publication.*

------------------------------------
REVIEWERS' COMMENTS
------------------------------------


Reviewer #1: 

In this manuscript, Woodward et al. examined the effects of pH on the pathogen Citrobacter rodentium. The authors found that pH mirroring different regions of the gastrointestinal tract differentially regulated virulence gene expression and cell morphology. The authors supported their findings with well-designed animal models. This work is very timely as the intestinal environment, including the pH, is not often assessed in animal models of infection. The manuscript is well-written, the figures are excellent and overall, the work is very compelling. The authors did an excellent job addressing the concerns of the original reviewers. As a new reviewer, I only have two minor comments.

*Thank you for taking the time to review our manuscript and provide thoughtful feedback, particularly regarding the supplementation of additional references which help to strengthen our findings.*

Comments:
1.    I am surprised to see a pH of 5.4 in the ileum. This pH is a bit lower compared to other stains of mice- particularly FvBN mice. I think Figure 1A needs to be modified to include more of the literature that measures intestinal pH along the length of the murine gastrointestinal tract in other animals strains. The current literature appears to only covers the pH of humans. Here are some pertinent references: PMID: 9662405, PMID: 24072680, PMID: 33670214, PMID: 18088506, PMID: 24429819, PMID: 10480906. The majority of these studies observed a pH~7 in the ileum. However, the data looks solid in the manuscript, and I trust that the authors are correct in reporting a pH of ~5 in the small intestine of the BALB/c mice.

*Thank you for highlighting relevant references for murine ileal pH. These values are all consistent with what was measured in the ileum of the C57BL/6 mice, regardless of diet, and we agree it is surprising that pH is lower in Balb/c mice. However, these values agree with measured intestinal pH as reported in PMID 18088506. We feel confident in our measurements of pH in this mouse background and believe this could be an important consideration in planning pH-dependent experiments in this mouse strain. We have now added several of these references to the text to strengthen the background regarding murine intestinal pH and have amended Figure 1A to include values reported in Wistar rats as well as the FVB/N and ICR murine background strains as well as BALB/c.*

2.    I think it's interesting that C. rodentium elevates the pH of the intestine, when the same effect is observed in humans in response to C. difficile infection (PMID: 25552580). This point could be added to the discussion to strengthen the paragraph on pH in humans.

*This is a very interesting point, thank you. While we expected to see a decrease in intestinal pH post-infection due to the high pathogen burden which we believed would acidify the intestinal content, we did not observe a significant change in pH. It is certainly possible that this is due to manipulation of proton transport by* C. rodentium *to preserve intestinal pH at favourable levels. We have rearranged the text slightly and added the following to the discussion on lines 600-605:*

“Surprisingly, downstream cecal pH did not change at peak infection, despite high pathogen burdens which were expected to acidify the gut content (Figure 6). Enteric pathogen *Clostridium difficile* has been shown to actively inhibit ion exchange to promote pathogen-favourable conditions, resulting in more alkaline stool in infected patients (61). It may be that *C. rodentium* is similarly able to manipulate ion transport at key niche locations, such as in the cecum, to maintain favourable conditions for pathogen expansion.”

------------------------------------


Reviewer #2: 

This paper has been reviewed before, so I am not going to repeat the general summary. It is well written and the discussion is informative. 

The original reviewers raised some concerns and the authors, in most parts, provided satisfactory responses. Nonetheless, the paper remains somewhat descriptive. 

The authors first determined the regional pH along the gut. They then conducted phenotypic and gene expression characterizations in the different pHs. However, the conclusions were not tested in vivo. In the last two figures the authors focused on the impact of colitis on the gastric pH. For me, Fig. 1-5 and Fig. 6-7 are disjointed, the paper seems to report two stories in one.  

Specific comments:

1.    The explanation of the observed increase in biomass at pH 3.5 in the absence of metabolic activity is not clear ("encapsulation of community members within biofilms").

*Thank you for this feedback, we have now expanded on this point to help explain why we would expect this increase in biomass. We have added the following to lines 298-303 of the text.*

*“Biofilm formation is an important stress-induced protective behaviour of bacteria which allows for the survival of community members shielded within. Aside from direct death of bacteria under low pH, bacteria within biofilms are also known to decrease their metabolic activity under stress, which promotes survival and persistence, especially as many stressors affect the bacterial membrane (such as antibiotics or pH) and have the greatest impacts on actively growing bacteria.”*

2.    While the authors tested cell attachment, they haven't quantified actin polymerization in CMT-93 cells. In their rebuttal they justified this by citing Liang et al, PNAS 2023. However, this paper reports actin straining in CMT-93 cells.

*Since CMT-93 cells do not induce visible actin polymerization in our experiments, we instead utilized Caco-2/TC7 cells, which allow for direct observation and quantification of pedestal formation. This approach also helped to address Reviewer 3’s comment of using another cells line to conduct pedestal quantification.*

*We have now performed microscopy assays on Caco-2/TC7 cells infected with* C. rodentium *pre-induced at pHs 7 (neutral pH) and 4.8 (the pH at which we observed induction of T3SS gene expression), staining for actin polymerization. In both cell lines we have now successfully shown increased pedestal formation (evidenced by increased hotspots of actin polymerization co-localized with bacteria) after pre-induction at pH 4.8 (Figure 2D).*

*While we did not expect to observe actin polymerization after infection, we repeated this assay with CMT-93 cells, and have also observed increased induction of pedestal formation following bacterial pre-induction at pH 4.8. However, the effect is much more subtle in this cell background (Figure S2I).*

*Together with our supplementary data showing decreased attachment to CMT-93 cells by the ∆escN T3SS-deficient* C. rodentium *strain (Figure S2)*, *we believe this data supports the induction of pH-dependent T3SS induction and attachment.*

3.    To translate the data to infection, it might be useful to test infection outcomes of C. rodentium grown in LB in different pH's.  Moreover, a kfc mutant should be tested in vivo.

*We thank the editor for their understanding in limiting the amount of additional animal experiments. While we have decided not to test a kfc mutant strain* in vivo, *we have attempted to test infection outcomes of* C. rodentium *pre-induced in LB at different pHs.*

*As described in response to our original reviews from mBio, we previously gavaged mice with* C. rodentium *pre-conditioned anaerobically at pH 7 and pH 5.7 before sacrifice 1 hour later, to harvest the entire intestinal tract for plating and enumeration of viable* C. rodentium.

*We ultimately decided to remove these data as we were not convinced this was the best way to demonstrate in vivo survival of acid adapted C. rodentium. Likewise, we agree with the reviewer that we cannot rule out the possibility that C. rodentium is actually dead rather than non-recoverable through this assay.*

*The 1 hour timepoint was initially chosen to avoid loss of C. rodentium via passage through the gut and excretion via the stool. The average intestinal transit time of a C57BL/6 mouse is approximately 4 hours. Likewise, gastric emptying time occurs on average between 30-120 minutes. Therefore, sacrifice at 1 hour post-inoculation was chosen to increase the likelihood that bacteria has passed from the stomach into the small intestine but had not yet been lost in the feces at the time of sampling.*

4.    In Figs. 3 and 4 the authors report expression of multiple genes across the pH range. While selecting few rationale targets, a transcriptomic approach would provide an unbiased readout. Moreover, the authors could probably use these data as a guide to generate specific mutants for in vivo testing. Otherwise, the relevance of the measurements remains elusive.

*While we were unable to run a transcriptomics experiment for the purposes of this publication, we did select gene targets associated with all known acid adaptation-related genes in the* C. rodentium *genome. This was done in response to our initial round of reviews which commented that we did not fully investigate acid adaptation genes by qPCR. The genes tested cover various known strategies of acid resistance including: amino acid decarboxylation, proton efflux, and both specific and non-specific stress response mechanisms.*

*We have now also created a* C. rodentium *∆*ldcC *deletion mutant to follow up on our observation that* ldcC *expression is induced by alterations to intestinal pH.* ldcC *was originally investigated in response to initial reviewer comments that we did not investigate the role of the gad system in acid regulation of* C. rodentium. *The gad system is important in related pathogenic* E. coli *species for acid tolerance, however* C. rodentium *does not encode homologous genes. The gad system does however represent a key strategy of bacteria to survive low pH - amino acid decarboxylation. To investigate whether* C. rodentium *may use a similar strategy, we looked for homology to other amino acid decarboxylation systems and identified* ldcC.

*While we have not pursued* in vivo *testing, to demonstrate the importance of* ldcC *to acid responses in* C. rodentium*, we have now performed a survival assay at gastric pH 3.5 to determine whether the* ∆ldcC *strain exhibits impaired viability under post-gastric conditions as compared to WT* C. rodentium *(Figure S3B)*. *We found that deletion of* ldcC *decreased* C. rodentium *viability following 120 minutes of exposure to gastric pH 3.5, suggesting that amino acid decarboxylation is an important acid response in* C. rodentium*.*

5.    The rationale for measuring changes to gastric pH and expression of host gene in response to C. rodentium infection is not apparent, considering that at days 4 and 8 pi C. rodentium is found further down the GI tract. Nonetheless, as the change in gastric pH seems to be a generic response to tissue damage, this is a novel discovery. However, the gastric data seem an add-on to the C. rodentium responses to a pH gradient described in earlier parts of the paper. The authors should better integrate these two "stories".

*Our group has previously demonstrated that* C. rodentium *colonizes the entire length of the GI tract at peak infection. While it is found at the highest burden in the large intestine, we have found that re-seeding of the upper intestinal tract by bacteria shed in the feces and subsequently re-ingested by coprophagy is important for the maintenance and seeding of upper intestinal regions (Woodward et al 2022, ISME J). For that reason we measured pH across the entire length of the intestinal tract at peak infection, including the stomach which is an under-investigated organ in enteric infectious disease research (despite the fact that all enteric pathogens must pass through the stomach). While downstream intestinal pH did not change as expected, the alteration of gastric pH post-infection has important implications for re-ingestion and intestinal re-seeding in mice, despite not being a region that is actively colonized by* C. rodentium*. We therefore believed it likely that altered gastric pH post-infection was the result of an altered host response rather than a manipulation by the pathogen and sought to investigate this further.*

*We have now further attempted to smooth the text transitions between the pathogen and host response data to help bridge these two facets which we believe are integrated and nicely illustrate the evolutionary arms race between pathogens and their hosts.*

6.  I thought the authors should have quantified shedding of the wild type, mutant and commensal E. coli strains in the last experiment.

*While we did not quantify fecal shedding over the 4-day infection in this study, we did quantify gut bacterial burden at the time of gastric pH measurement on day 4 which we believed was more relevant to our outputs. These data were originally included in Figure S6G, showing that bacterial burden of the* ∆escN *strain or Mt1B1 do not impact pH, unlike WT* C. rodentium*. We have now also included the cecum and colon colonization for each strain in Figure S6H (and below).*

C. rodentium ∆escN, WT *and* E. coli Mt1b1 *strains displayed similar levels of colonization at day 4 post-infection - this timepoint was strategically chosen so that bacterial colonization would be similar across all groups, as later post-inoculation* ∆escN *is quickly cleared from the gut. Fecal shedding data of* E. coli Mt1b1 *in our mouse model is previously reported by Serapio et al 2022 (*[*10.1016/j.celrep.2022.110731*](http://dx.doi.org/10.1016/j.celrep.2022.110731)*).*

------------------------------------

Reviewer #3: 

In this work Woodward et al study the effect of difference pH levels on the murine bacterial pathogen Citrobacter rodentium, a model of human attaching and effacing pathogens. They assessed the pH levels throughout the gastrointestinal tract and conducted various in vitro assays to evaluate bacterial responses under conditions that simulate the intestinal environment. They also determined host acid-related characteristics and responses. The topic is interesting and some of the observations, such as pH measurements across the GI tract, and host gastric pH under infection and inflammation are important and interesting and will definitely contribute the field. However, some of the major claims are overreaching and lack sufficient data support.

Comments:
Lines 232-241 - The major claim of growth arrest at pH 3.5 is well supported, but it is unclear to me how the authors distinguish growth and biofilm formation. Both assays appear to be reading absorbance at 600nm. Also, metabolic activity and biofilm production were measured after 48 hours of incubation, at which the bacteria are probably at deep stationary phase, although the transit time in the small intestine is a few hours. Cell morphology dramatically change much faster, as the authors show in Fig4. Other readouts were also taken much earlier.

*The reviewer brings up a valid point. Biofilm formation assays were performed by growing the bacteria statically for 48 hours in keeping with the standard for biofilm assays for related* E. coli (*Merritt, 2015 doi: 10.1002/9780471729259.mc01b01s00*)*.* *The OD600 measurement of the biofilm was performed specifically after removal of planktonic bacteria, and represents a relative quantification of bacteria adherent to the assay plate. In comparison, bacterial growth was evaluated in a plate reader by growing the bacteria with agitation for 20 hours, conditions which typically do not favour the formation of a biofilm.*

*Additionally, while we have used OD600 measurements to evaluate both biofilm and growth, we have also confirmed the lack of growth at pH 3.5 in the paper itself via the survival assays which demonstrate only a decrease in total bacteria over time at pH 3.5 and so we are confident in this conclusion.*

*Anecdotally we have further noted this lack of growth from plating the inoculum of our various epithelial cell infection assays, in which we noted no increase in bacteria from the amount inoculated at the start of the 3.5 hour subculture.*

Epithelial attachment - Actin rearrangements and pedestal formation are the hallmark of virulence for A&E pathogens. Although the escV mutant demonstrates reduced adherence to CMT-93 cells, highlighting the dependence on TTSS, it remains unclear why the authors did not utilize a cell line that would allow direct observation and quantification of pedestal formation.

*We have now performed microscopy assays on infected Caco-2/TC7 cells pre-induced at pHs 7 (neutral pH) and 4.8 (the pH at which we observed induction of T3SS gene expression), staining for actin polymerization. We have now successfully shown increased pedestal formation (evidenced by increased hotspots of actin polymerization co-localized with bacteria) after pre-induction at pH 4.8 (Figure 2D). Together with our supplementary data showing decreased attachment to CMT-93 cells by the ∆escN T3SS-deficient* C. rodentium *strain (Figure S2)*, *we believe that these data support the induction of pH-dependent T3SS induction.*

Bacterial stress responses - The claim that C. rodentium uses amino acid decarboxylation to combat acid induced stress is an overstatement, given the limited supporting data. Demonstrating reduced acid tolerance in a mutant LdcC strain would provide more convincing evidence for this claim.

*We have now created a* ∆ldcC *strain to test for acid stress. We have now run a survival assay to test the viability of the* ∆ldcC *at pH 3.5 compared to the WT* C. rodentium *over 120 minutes (the upper limit of gastric emptying time in humans reported in the literature). We have found that deletion of* ldcC *does impact* C. rodentium *viability at gastric pH, supporting the hypothesis that amino acid decarboxylation is a strategy to combat acid-induced stress as in related* E. coli *(Figure S3B). We believed this information was important to investigate as previous rounds of review had inquired after the fact that* C. rodentium *does not have genes homologous to the amino acid decarboxylation gad system of* E. coli *and related pathogen EHEC.*

*This new data has been included in Figure 3B.*

The increase in atpD expression in pH4.4 - it seems to mostly rely on 1 data point. An outlier test should be conducted here, and in Figure 4C (for degP).

*We have run an outlier test on both* atpD *and* degP *data and did not identify any outliers. We have amended the figure legend text to specify that an outlier test was run using the ROUT method (Q = 1%).*

*We have also included the* atpD *plot without this point below, which still demonstrates significance. However, we have also done the analysis with and without these points and found it does not impact the results. Stats were run on the log2 expression and not the fold change, as represented below.*

atpD *inclusive* p*-value 0.0056*

atpD *exclusive* p*-value 0.0219*

*Likewise, we did not identify any points as outliers for* degP *expression. As only 3 biological replicates were performed for* degP *activation, we are not able to perform statistics on the data excluding this replicate.*

Line 337 - unclear. What is "fluid regulation of membrane protein synthesis"?

*Thank you for this feedback, we have now attempted to reword the text for clarity. The following text has on lines 416-419 has been amended:*

*“The above data suggests that membrane protein synthesis and degradation must be tightly regulated to allow for successful pathogen adaptation to variable intestinal pH. If this is the case, interfering with low pH-induced membrane regulation will impact* C. rodentium *survival under acidic conditions.”*

Fig 6B - I'm not a statistician, but at a glance, there appears to be a low correlation between the data points and the shaded area. Same correlation in Fig. 7B looks convincing.

*The same statistics have been run on both plots (Spearman correlation;* p *= 0.007, rho = -4983). The issue with Figure 6B is that the CFU data should be plotted on a log scale so as not to be visually skewed towards very high values. Plotting it on a log scale allows for better visualization of the relationship between pH and CFU (see below).*

*However, including the uninfected control mice makes for an awkward visualization of the data. Therefore we have chosen to leave the plot as shown is in the paper (Figure 6B).*

*To better answer your question, we have also run this correlation excluding the uninfected controls. While the correlation is weaker and no longer significant (*p *= 0.059), the trend towards decreasing gastric pH with increasing pathogen colonization remains the same.*

Lines 452-454  - Bacterial burden should be quantified across the experimental groups by plating, qPCR or any other valid method.

*The bacterial burden at the point of pH measurement was quantified by plating for CFU. This data was originally included in Figure S6G as a correlation with gastric pH. We have now added an additional panel to this supplementary figure to show the colonization data alone (Figure S6H). While there is a difference in colonization levels between WT and* ∆escN C. rodentium *strains, unlike in WT colonization, there is no correlation between colonization levels and gastric pH at day 4 post-infection in either the* ∆escN *or* E. coli *Mt1B1 colonized mice. Therefore we believe this relationship is valid.*

------------------------------------

COMMENTS FROM THE ACADEMIC EDITOR:

1. The authors could consider bringing FigS1 C to the main Fig 1 as a new panel. Even if they don’t take this suggestion, the legend of this figure panel should be clarified regarding how many BL6 mice and how many BALBc mice. Currently is just says N=8-18 but does not state the sample size for each genetic background.

*Thank you for the suggestion, we have chosen to keep this panel in Fig S1C but have now included the sample size per genetic background.*

2. In the section host-adaptation, page 16 line 352, the authors should clarify if they mean that C. rodentium adapts evolutionary or if to physiological adaptation.

*We are referring specifically to physiological adaptation, particularly as it may relate to this ‘host-adaptive’ state of* C. rodentium *that has been previously described. This host-adapted and hyper-virulent state of* C. rodentium *which is observed in shed bacteria at peak infection, is known to be short-lived and lost following* ex vivo *passage (Wiles* et al *2005 doi:10.1111/j.1462-5822.2005.00544.x*).

*Thank you for the suggestion, we have now expanded on our introduction of* C. rodentium *host-adaptation and clarified this in the text as follows on lines 438-443:*

*“During an established infection,* C. rodentium *shed from the gut at peak infection is known to display a hypervirulent state characterized by altered T3SS expression and tissue colonization dynamics, and the ability to colonize new hosts at a reduced infectious dose, allowing for more effective transmission. This hypervirulent state is quickly lost following* ex vivo *passage, indicating that it results from within-host adaptation rather than evolutionary change (54,55).”*

3. In the section Gastric PH decreases in response to further infection, the authors should state why they chose to measure pH at day 4 and at day 8 as done in Fig 6A. I am not asking for more experiments just for a rational of the choice.

*We chose to limit the in vivo pH measurements for ethical reasons as this was an endpoint procedure of the mice and chose an early and peak infection timepoint (days 4 and 8 respectively).*

*Thank you for this suggestion, we have now amended the text as follows (edits to lines 476-481) :*

*“All the above experiments were done by subjecting* C. rodentium *to the range of intestinal pH found within the naïve murine gut. However, many global gut environmental changes occur during enteric infection. Therefore, we next sought to further profile intestinal pH at peak* C. rodentium *infection to determine if infection influences environmental pH, either as a result of pathogen colonization or an active host response. To test this, we measured gastric and cecal pH of infected mice at an early (day 4) and peak (day 8) infection timepoint and compared values to the corresponding pH in uninfected control mice.”*

__________________________________________________
In compliance with data protection regulations, you may request that we remove your personal registration details at any time.  (Use the following URL: <https://www.editorialmanager.com/pbiology/login.asp?a=r>). Please contact the publication office if you have any questions.
