# Supplementary material for: Both pathogen and host dynamically adapt pH responses along the intestinal tract during enteric bacterial infection
Source: PLoS Biol. 2024 Aug 15;22(8):e3002761. doi: 10.1371/journal.pbio.3002761 (PMC11349234; doi:10.1371/journal.pbio.3002761)
Supplement: Attachment — Submitted filename: Response-to-reviewers_editorial_requests.docx [file pbio.3002761.s013.docx]

Dear Dr Finlay,

Thank you for your patience while we considered your revised manuscript "Pathogen and host adapt pH responses during enteric infection" for publication as a Research Article at PLOS Biology. This revised version of your manuscript has been evaluated by the PLOS Biology editors and the Academic Editor.

Based on our Academic Editor's assessment of your revision, we are likely to accept this manuscript for publication, provided you satisfactorily address the remaining editorial points. Please also make sure to address the following data and other policy-related requests.

We thank the Editors for their careful consideration of our manuscript and have responded to the requests point-by-point below.

a) We routinely suggest changes to titles to ensure maximum accessibility for a broad, non-specialist readership, and to ensure they reflect the contents of the paper. In this case, we would suggest a minor edit to the title, as follows. Please ensure you change both the manuscript file and the online submission system, as they need to match for final acceptance:

"Both pathogen and host adapt dynamically to pH changes along the intestinal tract during enteric bacterial infection"

We have now adapted the title to read ‘Both pathogen and host dynamically adapt pH responses along the intestinal tract during enteric bacterial infection’. This minor alteration to the suggested title was done so as not to imply that the host is responding to pH changes along the intestinal tract. We have been able to show that pH changes to the gastric environment are a response to intestinal damage but not to changes to intestinal pH caused by infection. However, if this isn’t a suitable alteration we are happy to change it to the suggested title.

b) Given that some of the explanations given in the point by point response would be good for all readers to have access to, we would encourage that you opt into the transparent peer-review option, where editor letters and reviewer reports are published.

We wish to opt in to the transparent peer-review.

c) We do not allow Supplementary Materials and Methods, as well as Supplementary References. Please place your current Supplementary Materials and Methods, and their references, in the main methods section and main reference sections, respectively. The Supplementary Tables can remain there but they should be called out approapiately in the newly augmented "Materials and Methods" section.

Text from the original Supplemental Materials and Methods file have now been integrated into the main text and all references are now also accounted for in the main text.

d) Thank you for supplying the numerical values of most of the figures. However, we are missing the values for Figures SB3 and S6H. We have also noted that the data for Figure S4B seems to be mislabelled.

Apologies, the missing values have now been included and the labelling of Figure S4B has been corrected. Thank you for catching that for us.

e) Please note that per journal policy, the model system/species (mouse) studied should be clearly stated in the abstract of your manuscript. Thank you for already providing the name of Citrobacter rodentium.

We have now updated the abstract to specifically mention mice.

f) Please ensure that your Data Statement in the submission system accurately describes where your data can be found and is in final format, as it will be published as written there.

We have now updated the Data Statement.

g) Per journal policy, if you have generated any custom code during the course of this investigation, please make it available without restrictions upon publication. Please ensure that the code is sufficiently well documented and reusable, and that your Data Statement in the Editorial Manager submission system accurately describes where your code can be found.

Please note that we cannot accept sole deposition of code in GitHub, as this could be changed after publication. However, you can archive this version of your publicly available GitHub code to Zenodo. Once you do this, it will generate a DOI number, which you will need to provide in the Data Accessibility Statement (you are welcome to also provide the GitHub access information). See the process for doing this here: <https://docs.github.com/en/repositories/archiving-a-github-repository/referencing-and-citing-content>

We have not generated any custom code for the purposes of this manuscript.

As you address these items, please take this last chance to review your reference list to ensure that it is complete and correct. If you have cited papers that have been retracted, please include the rationale for doing so in the manuscript text, or remove these references and replace them with relevant current references. Any changes to the reference list should be mentioned in the cover letter that accompanies your revised manuscript.

We expect to receive your revised manuscript within two weeks. 

To submit your revision, please go to <https://www.editorialmanager.com/pbiology/> and log in as an Author. Click the link labelled 'Submissions Needing Revision' to find your submission record. Your revised submission must include the following:
-  a cover letter that should detail your responses to any editorial requests, if applicable, and whether changes have been made to the reference list
-  a Response to Reviewers file that provides a detailed response to the reviewers' comments (if applicable, if not applicable please do not delete your existing 'Response to Reviewers' file.)
-  a track-changes file indicating any changes that you have made to the manuscript. 

NOTE: If Supporting Information files are included with your article, note that these are not copyedited and will be published as they are submitted. Please ensure that these files are legible and of high quality (at least 300 dpi) in an easily accessible file format. For this reason, please be aware that any references listed in an SI file will not be indexed. For more information, see our Supporting Information guidelines:
<https://journals.plos.org/plosbiology/s/supporting-information>  

*Published Peer Review History*
Please note that you may have the opportunity to make the peer review history publicly available. The record will include editor decision letters (with reviews) and your responses to reviewer comments. If eligible, we will contact you to opt in or out. Please see here for more details:
<https://blogs.plos.org/plos/2019/05/plos-journals-now-open-for-published-peer-review/>

*Press*
Should you, your institution's press office or the journal office choose to press release your paper, please ensure you have opted out of Early Article Posting on the submission form. We ask that you notify us as soon as possible if you or your institution is planning to press release the article.

*Protocols deposition*
To enhance the reproducibility of your results, we recommend that if applicable you deposit your laboratory protocols in protocols.io, where a protocol can be assigned its own identifier (DOI) such that it can be cited independently in the future. Additionally, PLOS ONE offers an option for publishing peer-reviewed Lab Protocol articles, which describe protocols hosted on protocols.io. Read more information on sharing protocols at <https://plos.org/protocols?utm_medium=editorial-email&utm_source=authorletters&utm_campaign=protocols>


Please do not hesitate to contact me should you have any questions.

Sincerely,

Melissa

Melissa Vazquez Hernandez, Ph.D.
Associate Editor
[mvazquezhernandez@plos.org](mailto:mvazquezhernandez@plos.org)
PLOS Biology

__________________________________________________
In compliance with data protection regulations, you may request that we remove your personal registration details at any time.  (Use the following URL: <https://www.editorialmanager.com/pbiology/login.asp?a=r>). Please contact the publication office if you have any questions.
